# The effect of cold acclimation, deacclimation and reacclimation on metabolite profiles and freezing tolerance in winter wheat

**DOI:** 10.3389/fpls.2022.959118

**Published:** 2022-08-15

**Authors:** Gabija Vaitkevičiūtė, Andrius Aleliūnas, Yves Gibon, Rita Armonienė

**Affiliations:** ^1^Lithuanian Research Centre for Agriculture and Forestry, Institute of Agriculture, Akademija, Lithuania; ^2^Univ. Bordeaux, INRAE, Bordeaux Metabolome, UMR 1332 BFP, Villenave d’Ornon, France

**Keywords:** carbohydrate metabolism, climate change, cold stress, shoot biomass growth, *Triticum aestivum* L., winter hardiness

## Abstract

Global climate change will cause longer and warmer autumns, thus negatively affecting the quality of cold acclimation (CA) and reducing the freezing tolerance (FT) of winter wheat. Insufficient FT and fluctuating temperatures during winter can accelerate the deacclimation (DEA) process, whereas reacclimation (REA) is possible only while the vernalization requirement is unfulfilled. Six winter wheat genotypes with different winter hardiness profiles were used to evaluate the impact of constant low-temperature (2°C) and prolonged higher low-temperature (28 days at 10°C followed by 2°C until day 49) on shoot biomass and metabolite accumulation patterns in leaf and crown tissues throughout 49 days of CA, 7 days of DEA, and 14 days of REA. The FT of winter wheat was determined as LT_30_ values by conducting freezing tests after CA, DEA, and REA. Shoot biomass accumulation, projected as the green leaf area (GLA), was investigated by non-destructive RGB imaging-based phenotyping. Dynamics of carbohydrates, hexose phosphates, organic acids, proteins, and amino acids were assessed in leaf and crown tissues. Results revealed that exposure to higher low-temperature induced higher accumulation of shoot biomass and had a negative impact on FT of winter wheat. Prolonged higher low-temperature negatively affected the accumulation of soluble carbohydrates, protein content and amino acids, and had a positive effect on starch accumulation in leaf and crown tissues after CA, in comparison with the constant low-temperature treatment. DEA resulted in significantly reduced FT. Lower concentrations of glucose-6-phosphate, sucrose and proline, as well as higher concentrations of starch in leaves and crowns were found after DEA. The majority of the genotypes regained FT after REA; higher concentrations of glucose and malate in leaves, and sucrose in crown tissue were observed, whereas starch accumulation was decreased in both tissues. Negative correlations were determined between FT and starch concentration in leaves and crowns, while proline and proteins, accumulated in crowns, showed positive correlations with FT. This study broadens the knowledge regarding the effect of different low-temperature regimes on the dynamics of metabolite accumulation in winter wheat throughout CA, DEA, and REA, and its relationship to biomass accumulation and FT.

## Introduction

Global climate change poses a significant threat to the winter survival and grain yield of winter crops in temperate regions. The global average temperature is predicted to increase by up to 3.5°C during the next 80 years compared to pre-industrial times (IPCC, 2021). Prolonged and warmer autumns, followed by the exposure to numerous abiotic stresses throughout the winter season will negatively affect the long-term survival of winter type crops (Dalmannsdottir et al., 2017; Jørgensen et al., 2020). Moreover, increased clouding will lower the efficacy of photosynthesis, and decreased snow cover will expose the crops to freezing temperatures that might lead to winterkill (Rapacz et al., 2014; Kersebaum, 2022). The loss of snow insulation is extremely detrimental in combination with winter temperature fluctuations. As warmer spells are followed by sudden low negative temperatures, such cases result in yield loss as high as 90% (Armonienė et al., 2013; Vico et al., 2014). Therefore, breeding for adaptive stress-tolerant varieties of winter crops will be vital to meet the growing demand for food production.

Winter wheat is sown in autumn and possesses a vernalization requirement that is mandatory for the transition from vegetative to reproductive phase. The overwintering feature of winter wheat allows a prolonged vegetation period, and ultimately results in more than 30% grain yield increase as compared to spring wheat (Entz and Fowler, 1991; Fowler, 2012). The ability to withstand biotic and abiotic stresses during the cold season is known as winter hardiness, which consists of a complex of elements. One of the major components of winter hardiness is freezing tolerance (FT), and an inadequate FT in winter wheat limits the grain yield potential in the temperate regions (Fowler and Limin, 1997). FT in winter type crops is gained during the cold acclimation (CA) process. The process of CA occurs through a 28–56 day period of low positive temperatures within the range of 0–10°C in autumn (Fowler et al., 1996; Laudencia-Chingcuanco et al., 2011). CA can only be induced when the threshold induction temperature is reached. The threshold temperature for CA is widely accepted to be approximately 10°C, however, it varies between different species and varieties of winter crops (Fowler, 2008). Throughout CA, the growth of winter wheat is reduced, and numerous changes in transcriptome and metabolome are induced (Zhao Y. et al., 2019; Aleliūnas et al., 2020). Moreover, low autumn temperatures lead to various physiological changes, such as reduced shoot biomass growth and leaf elongation rate, and decreased tissue water content (Dalmannsdottir et al., 2017; Rys et al., 2020; Jaškūnė et al., 2022). Currently, the most documented conserved molecular signaling pathway, activated by CA, is the ICE-CBF-COR pathway. Low temperature promotes the biosynthesis of transcription factors (TFs), known as Inducers of CBF Expression (ICE), which upregulate C-Repeat Binding Factors (CBFs). Subsequently, CBFs induce the accumulation of cold-responsive (COR) proteins, involved in stress response and metabolism (see review by Liu et al., 2019). Low temperature stress also leads to decreased activity of temperature-sensitive enzymes and thus results in reduced photosynthesis (Savitch et al., 1997). A group of TFs and other regulatory proteins, which accumulate during cold stress, were likewise shown to negatively affect growth in *Arabidopsis thaliana* (Lantzouni et al., 2020).

A complex of factors, such as temperature, light intensity, and photoperiod, affect the CA of winter type plants (Rapacz et al., 2014). Due to global climate change, CA will be postponed until late autumn or early winter, while the shortened photoperiod will lead to insufficient accumulation of metabolites, ultimately, resulting in decreased FT and winterkill. However, low temperature is the main CA-inducing factor (Fowler et al., 1999), and detailed research is required on the effect of increasing temperatures upon the processes of CA and FT in crops. Furthermore, temperature fluctuations during winter are predicted to prematurely induce deacclimation (DEA), rendering the crops vulnerable to sudden cold spells. It is particularly dangerous in the second half of winter when vernalization saturation is already achieved. Therefore, DEA is becoming an increasingly important factor in the survival of winter type crops under different climate change scenarios (Rapacz et al., 2022). The return of freezing temperatures after sudden warmth is less detrimental to crops, which can reacclimate (REA) rapidly. It is known, that winter wheat is able to achieve REA if the vernalization saturation is unfulfilled, however, once the plants transit into the reproductive stage, REA is substantially limited (Gusta and Fowler, 1976; Mahfoozi et al., 2001). It has been proposed that the complex interaction between vernalization requirement, dehydrin proteins and soluble carbohydrates could play a role in REA (Vítámvás and Prášil, 2008; Trischuk et al., 2014). Several studies have investigated DEA and REA at the molecular level in woody plants (Yazdanpanah et al., 2021; Kwon et al., 2022), as well as *Triticum* and *Brassica* species (Vítámvás and Prášil, 2008; Trischuk et al., 2014; Horvath et al., 2020). Nevertheless, research is relatively scarce on the molecular mechanisms behind DEA and especially REA in diverse plant species and genotypes (Vyse et al., 2019).

While the number of studies regarding CA has been rising, investigation of the molecular processes behind DEA and REA will significantly contribute to improved adaptability of winter type crops in future breeding efforts under global climate change. However, research on metabolite accumulation in several tissues throughout multiple sampling points during CA, DEA, and REA can be time- and resource-consuming. Moreover, it is important to select a number of genotypes that display differences in gene expression and metabolite accumulation, as well as a wide range of phenotypes under identical environmental conditions (Kumar et al., 2017; Shamloo et al., 2017). The aim of this study was to assess the effect of prolonged elevated temperature during CA on shoot biomass accumulation, FT and metabolite accumulation within the leaf and crown tissues of 6 winter wheat genotypes throughout the stages of CA, DEA, and REA. The dynamics of metabolites in leaf and crown tissues during a 70-day experiment, comprised of CA, DEA, and REA phases, were investigated and the metabolite profiles between two low-temperature treatment groups and tissues were analyzed. The relationships between shoot biomass, FT and metabolites were likewise determined.

## Materials and methods

### Plant material, experimental design, and growth conditions

**FIGURE 1:**
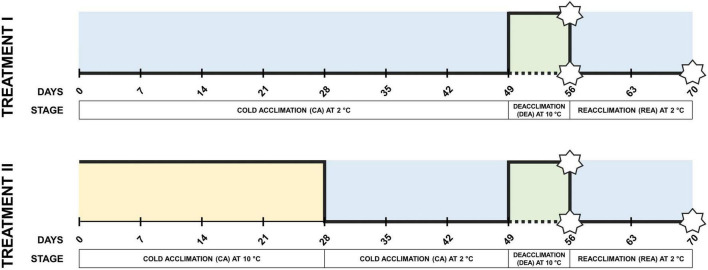
Experimental design of two different low-temperature treatments during CA. Stars are used to indicate freezing tests of winter wheat performed after CA, DEA, and REA. Dotted lines indicate the subgroups which have not gone through 7 days of DEA, and thus, are fully acclimated at 56 days instead (CA).

Six winter wheat varieties (“Hanswin”—Switzerland; “KWS Ferrum,” “Nordkap”—Germany; “SW Magnifik”—Sweden; “Lakaja DS,” “Sedula DS”—Lithuania) with different winter hardiness profiles under Lithuanian climate conditions (presented in order from lowest to highest) were selected (Jaškūnė et al., 2022). Seeds were imbibed on moist filter paper in Petri dishes and kept in the dark for 4 days at 4°C to stratify, and then transferred to room temperature for 16 h. A total of 216 seeds of each genotype were then sown in peat moss substrate (Durpeta, Lithuania) in 125 cm^3^ 28-well trays in a randomized pattern and grown in a greenhouse at 18°C temperature and 12 h photoperiod until the three-leaf stage was reached. Subsequently, the seedlings were transferred to a phytotron (PlantMaster, CLF Plant Climatics GmbH, Germany) and subjected to two different low-temperature treatment groups of CA (Figure 1). In treatment I, the plants were subjected to constant CA at 2°C for 49 days, while in treatment II the plants went through CA at 10°C for 28 days, followed by 2°C for 21 days. These temperatures were chosen to represent the commonly applied (2°C) and upper-limit (10°C) of CA-inducing temperatures under controlled conditions (Fowler et al., 1996; Fowler, 2008). CA in both treatment groups was followed by identical conditions of DEA at 10°C for 7 days, and REA at 2°C for 14 days. An additional subgroup within each treatment group was not subjected to DEA and instead was held at 2°C for additional 7 days of CA. Twelve-hour photoperiod, light intensity of 200 μmol m^–2^ s^–1^ and 80% relative air humidity were applied throughout the entire experiment in both treatment groups. All trays were watered regularly to keep the substrate moist.

### Sample collection and processing for metabolite assays

Samples of winter wheat leaf and crown tissues were collected before CA at time-point 0, and every 7 days henceforth during CA, DEA, and REA, always in the middle of the photoperiod. Three biological replicates were collected for each genotype and treatment group at each time-point. Each biological sample consisted of 3 individual plants pooled together. The samples were instantly submerged in liquid nitrogen and ground using the Mixer Mill MM 400 (Retsch, Germany). 50 and 20 mg fresh weight (FW) aliquots of leaf and crown tissue samples, respectively, were weighed into 1.1 ml Micronic tubes and stored at −80°C.

### Metabolite assays

Ethanolic fractionations of leaf and crown samples were carried out twice with 80% (v/v) EtOH—HEPES/KOH 10 mM (pH6) and once with 50% (v/v) EtOH—HEPES/KOH 10 mM (pH6), with a final volume of 650 μl (Geigenberger et al., 1996). The process was performed using a Microlab STAR automated liquid handling platform (Hamilton Robotics, Switzerland). Supernatants of leaf extracts were immediately used to measure chlorophyll a (chl a) and b (chl b), as well as total chlorophyll (total chl) content (Arsovski et al., 2018).

Glucose, fructose, sucrose (Geigenberger et al., 1996), glucose-1-phosphate (G1P), glucose-6-phosphate (G6P), fructose-6-phosphate (F6P) (Gibon et al., 2002), malate (Nunes-Nesi et al., 2007), polyphenols (Stevanato et al., 2004), total amino acids (Bantan-Polak et al., 2001), proline (Magné and Larher, 1992), and total antioxidant capacity (TAC) (Re et al., 1999) were determined in leaf and crown tissue extracts. Citrate was measured in crown extracts according to the producer’s instructions (Megazyme, Ireland). Pellets of both leaf and crown extracts were resuspended in 0.1 M NaOH and used to determine protein (Bradford, 1976) and starch (Hendriks et al., 2003) contents. Subsequently, they were washed twice with 0.5 M NaOH and lyophilized with a BenchTop Pro freeze dryer (SP Industries, United States). Remaining pellet weight (CWC), largely comprised of polysaccharides, such as cellulose and hemicellulose, was measured using a 1702 analytical balance (Sartorius AG, Germany). Assays were automated using the Microlab STARlet automated liquid handling platform (Hamilton Robotics, Switzerland). Metabolite measurements were carried out using the MP96 microplate reader (Safas, Monaco). Amino acid content was measured using the Xenius microplate fluorescence reader (Safas, Monaco). All biochemical assays and measurements were conducted at the HiTMe facility at Bordeaux-Metabolome, France.

### Determination of shoot biomass accumulation

The imbibed seeds of 6 winter wheat genotypes were sown in 1.5 dm^3^ pots with peat moss substrate in 3 biological replications, and grown in a greenhouse at 18°C and 12 h photoperiod until the three-leaf stage was reached. Then the plants were transferred to the phytotron and subjected to the same two CA treatment groups as described previously. All pots were watered regularly to keep the substrate moist. Starting from time-point 0 (pre-CA), images of winter wheat plants were taken every 7 days with a digital single lens reflectance (DSLR) camera EOS 2000D (Canon, United States) and a 18–55 mm lens. The open-source camera controlling software digiCamControl^1^ was used to automate the imaging process. Every individual plant was photographed at 90°, 180°, 270°, and 360° angles. Images were processed and projected green leaf area (GLA) was measured as a parameter of shoot biomass *via* ImageJ (Schneider et al., 2012). The average GLA value of each individual plant at each sampling point was calculated. After 70 days, the plants were cut above the crown region and their fresh weight was measured. The material was then dried at 55°C for 3 days in a Memmert INE 500 Precision Incubator (GmbH and Co., KG, Germany) and weighed to evaluate the dry biomass.

### Freezing tolerance tests

Each genotype was sown in 4 replicates for each target temperature, treatment group, and stage of acclimation in a randomized pattern. Each replicate was comprised of 10 imbibed seeds of each winter wheat genotype, which were sown into a single well (125 cm^3^) of a 28-well tray, containing peat moss substrate. Seedlings were grown in a greenhouse at 18°C and 12 h photoperiod until the three-leaf stage was reached, transferred to the phytotron and subjected to the same two CA treatment groups as described previously. Freezing tests were conducted after CA for 56 days, DEA for 7 days, and REA for 14 days (Figure 1). The plants were counted, and the trays were drenched with cold water prior to each freezing test. Freezing tests were carried out in the freezing chamber PE 2412 UY-LX (Angelantoni Industrie, Italy). The temperature of the substrate at crown depth was recorded at 2 min intervals using thermocouple probes and the KD7 data logger (Lumel, Poland). Over 6 h, the temperature was gradually decreased from 2 to −6°C and held until the substrate temperature stabilized. Subsequently, the temperature was decreased to target temperature (−12, −14, −16, and −18°C) at the rate of 1°C/h and held at each target temperature for 11 h. Following the freezing test, plants were removed and relocated to the phytotron, where they were kept at 2°C for 12 h in the dark. Leaves were cut 2 cm above the crown and plants were transferred to the greenhouse at 18°C. After 3 weeks, the numbers of regrown and dead plants were recorded. R package “MASS” was used to calculate LT_30_ (temperature, at which 30% of plants die) (Venables and Ripley, 2002).

### Statistical analyses

Statistical analyses were conducted using R v. 4.1.1 (R Core Team, 2022). Normality of data was tested using Shapiro–Wilk test. Unpaired t, ANOVA and *post hoc* Tukey’s HSD tests were applied for normally distributed data using the R package “agricolae” (de Mendiburu and Yaseen, 2020). Wilcoxon rank-sum and Kruskal-Wallis *H*-tests were applied for non-normally distributed data. Spearman’s Rank Correlation Coefficient was used to investigate the correlations between GLA, LT_30_ and metabolite content. PCA variable correlation plots were drawn using the R package “FactoMineR” (Lê et al., 2008). Heatmaps and PCA analyses were carried out on MetaboAnalyst v. 5.0 (Pang et al., 2021); data were normalized by median, transformed by cube root and scaled by applying the Pareto method.

## Results

### The effect of different low-temperature regimes during cold acclimation on shoot biomass accumulation of winter wheat

**FIGURE 2:**
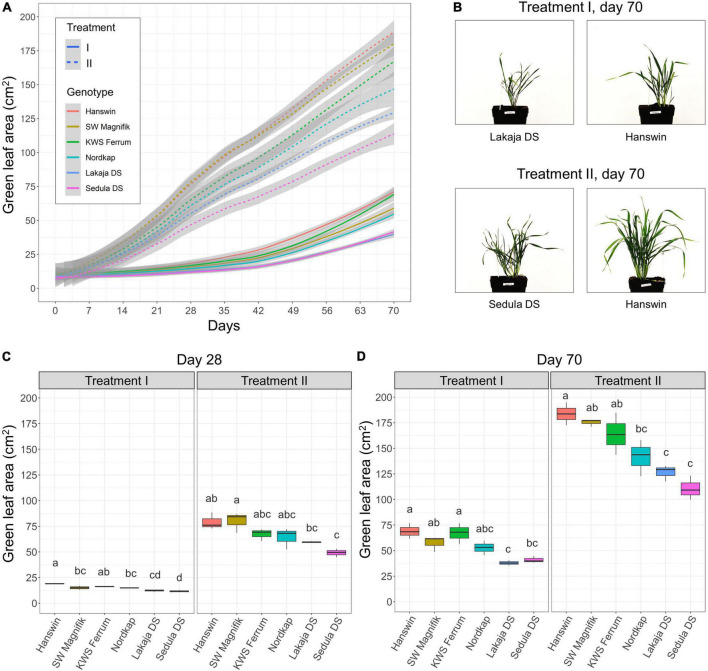
The effect of different low-temperature regimes during CA on shoot biomass accumulation of winter wheat. Dynamics of shoot biomass accumulation of 6 winter wheat genotypes during 49 days of CA, followed by 7 days of DEA and 14 days of REA in two low-temperature treatment groups. In treatment I, the plants were subjected to constant CA at 2°C for 49 days, while in treatment II the plants went through CA at 10°C for 28 days, followed by 2°C for 21 days. The gray areas indicate the 95% confidence interval **(A)**. Side-view images of the wheat genotypes, displaying least and most shoot biomass growth, subjected to treatments I and II, on day 70 **(B)**. Shoot biomass of 6 winter wheat genotypes on day 28 **(C)** and day 70 **(D)** in treatments I and II. The letters above the boxplots indicate significant (*p* < 0.05) differences between genotypes within each treatment group.

To evaluate the effects of a constant low-temperature treatment and a prolonged elevated low-temperature treatment during CA on shoot biomass accumulation of winter wheat, projected GLA was measured throughout the experiment (Figure 2). Plant exposure to elevated low-temperature during the first 28 days of CA resulted in increased shoot biomass accumulation in all 6 winter wheat genotypes for the duration of the experiment (*p* < 0.001), compared to plants in the constant low-temperature treatment (Figure 2 and Supplementary Table 1). The average GLA values ranged from 14.6 in treatment I to 66.5 cm^2^ in treatment II after 28 days of CA (Figure 2C and Supplementary Table 1). Lowering of temperature to 2°C after 28 days of CA did not slow down the accumulation of shoot biomass, and GLA was significantly higher in comparison with the slow biomass accumulation pattern of wheat plants under treatment I until the end of the experiment (Figure 2A and Supplementary Table 1). Furthermore, the genotype had a significant (*p* < 0.01) effect on shoot biomass accumulation after CA (day 49), DEA (day 56), and REA (day 70) in both treatment groups (Supplementary Table 2). Projected GLA of winter wheat genotypes ranged from 37.9 to 68.9 cm^2^ in “Lakaja DS” and “Hanswin” after 70 days in treatment I, respectively. Treatment II resulted in “Sedula DS” accumulating the least shoot biomass, yielding GLA value of 110.73, whereas “Hanswin” generated the most shoot biomass, as indicated by a GLA of 183.66 cm^2^ (Figures 2B,D and Supplementary Table 2). Projected GLA values showed highly significant Pearson’s correlations (*p* < 0.001) with fresh and dry biomass of wheat plants measured at the end of the experiment, *r* = 1 and *r* = 0.98, respectively, thus confirming the reliability of image-based shoot biomass assessment.

### Effect of different low-temperature treatments on freezing tolerance of cold acclimation, deacclimation, and reacclimation winter wheat

**FIGURE 3:**
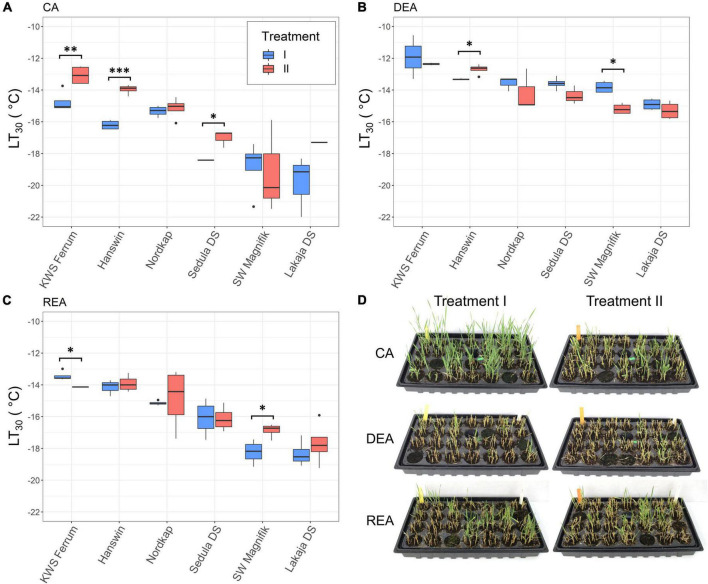
The effect of different low-temperature treatments during CA on FT of 6 winter wheat genotypes at stages of CA **(A)**, DEA **(B)**, and REA **(C)**. In treatment I, the plants were subjected to constant CA at 2°C for 49 days, while in treatment II the plants went through CA at 10°C for 28 days, followed by 2°C for 21 days. Images of winter wheat, regrown after –16°C freezing tests, at stages of CA, DEA and REA **(D)** * indicates significant differences at *p* < 0.05, ^**^ at *p* < 0.01, and ^***^ at *p* < 0.001.

Exposure to a period of higher temperature during CA had a significant effect on FT of winter wheat after 56 days of CA (*p* < 0.01) (Figure 3 and Supplementary Table 3). After CA, treatment II resulted in significantly (*p* < 0.05) reduced FT of the genotypes “KWS Ferrum,” “Hanswin,” and “Sedula DS,” compared to plants subjected to constant CA at 2°C. The LT_30_ values of these genotypes were higher by 1.67, 2.23, and 1.4°C, respectively (Figure 3A). Furthermore, DEA significantly (*p* < 0.05) reduced the FT of “KWS Ferrum,” “Hanswin,” “Sedula DS,” and “SW Magnifik” in treatment II, whereas in treatment I it resulted in decreased FT of all 6 genotypes. The majority of genotypes had the capacity to regain FT (*p* < 0.05) after REA, as compared with DEA (Supplementary Figure 1).

Genotype, as a statistical factor, had a significant (*p* < 0.001) effect on FT in both low-temperature treatments at all 3 stages of acclimation (Supplementary Table 3). “KWS Ferrum” and “Hanswin” were the most freezing-susceptible genotypes, whereas “SW Magnifik” and “Lakaja DS” remained the most freezing-tolerant genotypes. This tendency was maintained in both treatment groups throughout CA, DEA, and REA (Supplementary Table 4).

### Separation of metabolite profiles between winter wheat leaf and crown tissues during cold acclimation, deacclimation, and reacclimation

The metabolic traits showed a complete separation by tissue into two distinct clusters in both treatment groups throughout the whole experiment. PC1 explained 71, 84.2, 78.6, and 82.2% of the variance in treatment I, and 71, 84.2, 83.8, and 83.2% of the variance in treatment II on days 0, 49, 56, and 70, respectively (Supplementary Figure 2). The leaves in treatment I accumulated significantly lower concentrations of amino acids, proline, F6P, fructose, G1P, G6P, glucose, and malate compared to the crowns (*p* < 0.05), however, the leaves contained more polyphenols, protein content, and TAC (*p* < 0.05) throughout all stages of acclimation (Supplementary Figure 3 and Supplementary Table 5). Treatment II induced the same pattern of metabolites between the two tissues (*p* < 0.05). Moreover, in treatment II, higher levels of CWC were observed in the leaves (*p* < 0.05), whereas the concentration of sucrose was lower compared to the crowns (*p* < 0.001) throughout all stages of CA (Supplementary Figure 4 and Supplementary Table 6). Citrate was found to be below the detection threshold in the majority of leaf samples and therefore was assessed only in crowns.

### Differences in temporal metabolite profile dynamics between different low-temperature treatments in leaf and crown tissues during cold acclimation, deacclimation, and reacclimation

Major metabolic traits measured in winter wheat leaf and crown tissues were affected by elevated low-temperature during CA (Supplementary Figure 5). Principal component analyses (PCAs) of leaf metabolite profiles revealed a clear separation of treatment groups after 7 days of CA, in which the first principal component (PC1) explained 55.7% of the variance. The separation at this time-point was weaker in crown tissue, where PC1 was responsible for 38.7% of the total observed variance. The analyses confirmed the tendency of treatments I and II to separate in both tissues on day 49 of CA, as well as after DEA and REA (Supplementary Figure 5).

**FIGURE 4:**
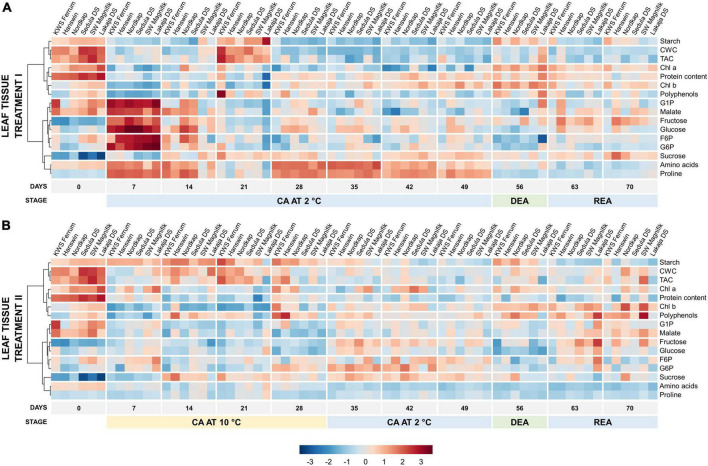
Evolution of major biomass components and sugar-phosphates in the leaves of 6 winter wheat genotypes during two CA, DEA, and REA treatments. Heatmaps of metabolite concentrations in treatment I **(A)** and treatment II **(B)**. Each column represents the results of a single genotype on a given time-point. The genotypes are ordered by FT, from lowest to highest (“KWS Ferrum,” “Hanswin,” “Nordkap,” “Sedula DS,” “SW Magnifik,” “Lakaja DS,” respectively). Each row represents the scaled concentration of a given metabolite; a single cell signifies the average value of three biological replicates. Low values are marked as blue, and high values are marked as red. The data of both treatments I and II were treated and analyzed simultaneously and are intercomparable.

**FIGURE 5:**
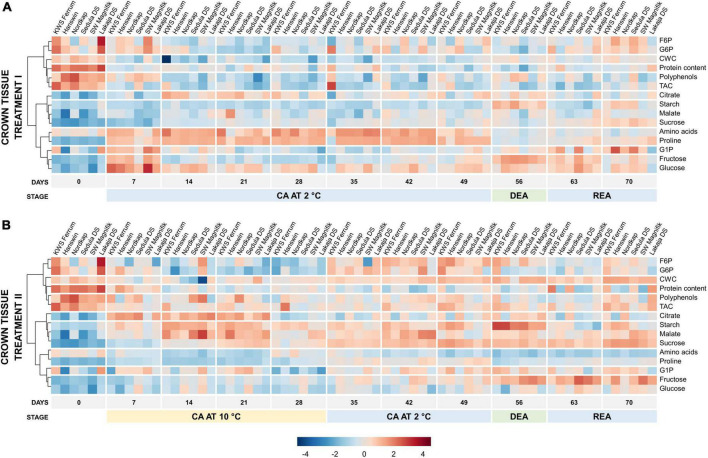
Evolution of major biomass components and sugar-phosphates in the crowns of 6 winter wheat genotypes during two CA, DEA, and REA treatments. Heatmaps of metabolite concentrations in treatment I **(A)** and treatment II **(B)**. Each column represents the results of a single genotype on a given time-point. The genotypes are ordered by FT, from lowest to highest (“KWS Ferrum,” “Hanswin,” “Nordkap,” “Sedula DS,” “SW Magnifik,” “Lakaja DS,” respectively). Each row represents the scaled concentration of a given metabolite; a single cell signifies the average value of three biological replicates. Low values are marked as blue, and high values are marked as red. The data of both treatments I and II were treated and analyzed simultaneously and are intercomparable.

Heatmaps showed treatment-induced differences in temporal metabolic profiles of leaf and crown tissues throughout the experiment (Figures 4, 5). Prior to CA (day 0), the leaves contained high levels of starch, cell wall content, chl a and proteins (Figure 4A). On day 7 of constant CA at 2°C (treatment I), these levels were reduced, whereas G1P, malate, fructose, glucose, F6P, G6P strongly increased, and amino acids and proline were much less affected (Supplementary Figure 6). By the 21st day of CA, the concentrations of these metabolites had decreased while starch, CWC and TAC were strongly increased. Over the remaining days of CA at 2°C (days 28–49), leaves switched to the accumulation of amino acids and proline. DEA (day 56) at 10°C in treatment I resulted in the concentrations of leaf chl a, total chl and starch to increase, and the concentrations of amino acids, F6P, G6P, proline, and sucrose to decrease significantly (*p* < 0.05), in comparison with CA (Supplementary Table 7). After REA, malate and proline increased while starch decreased, all significantly (*p* < 0.05) in comparison with DEA, while the concentrations of F6P, G6P, glucose, and sucrose were able to reliably (*p* < 0.05) return to CA levels.

Prolonged higher low-temperature during CA resulted in an altered temporal metabolic profile of winter wheat leaves. Notably, amino acids and proline were significantly (*p* < 0.001) lower under treatment II throughout the entire experiment (Figure 4B and Supplementary Figure 6). The concentrations of G1P, malate, fructose, glucose, F6P, and G6P were significantly lower compared to treatment I (*p* < 0.001) on day 7 of CA. However, at this time-point, increased temperature resulted in significantly increased levels of starch, chl a, protein content, and polyphenols (*p* < 0.01) (Supplementary Figure 6 and Supplementary Table 8). The concentration of starch remained high throughout the remaining 21 days of CA at 10°C. After the temperature in treatment II was reduced to 2°C on days 35–49, the levels of starch declined, and only relatively weak increases in G1P, fructose, glucose, F6P, G6P, and sucrose were recorded (Figure 4B). Treatment II resulted in lower protein content (*p* < 0.001), and higher concentrations of starch, CWC, TAC, chl a, polyphenols, F6P, and G6P (*p* < 0.05) after 49 days of CA, compared with treatment I. Following DEA, the concentrations of protein content, fructose and glucose were significantly lower (*p* < 0.05), and the concentrations of CWC, chl a, polyphenols, G1P, malate, F6P, and G6P were significantly higher (*p* < 0.05) in treatment II. After REA, treatment II was shown to induce lower concentrations of starch, protein content, and sucrose (*p* < 0.05), and higher concentrations of CWC, TAC, and polyphenols (*p* < 0.01) (Supplementary Figure 6 and Supplementary Table 8). In comparison to CA, DEA in treatment II showed significant increases of chl a and starch, and significant decreases (*p* < 0.05) of F6P, G6P, fructose, glucose, malate, proline and sucrose (Supplementary Table 7). After REA, the concentrations of malate and amino acids significantly increased and decreased, respectively, while the concentrations of Chl a, fructose, glucose, and starch under higher low-temperature conditions were returned to CA levels (*p* < 0.05).

The changes in metabolic traits were less distinctive in crown tissue (Figure 5). On day 0, non-acclimated crowns contained high concentrations of proteins, polyphenols and TAC. After 7 days of CA, treatment I induced a strong increase of amino acids, proline, G1P, fructose, and glucose concentrations (Figure 5A). Over the remaining CA (days 14–49), the concentrations of amino acids and proline remained high (Supplementary Figure 7). DEA resulted in significantly higher concentrations of fructose and starch, and significantly lower (*p* < 0.05) concentrations of amino acids, F6P, G6P, proline, and sucrose in crown tissues of treatment I, when compared with CA (Supplementary Table 9). Following REA, crown starch and sucrose concentrations significantly increased, and citrate, polyphenol and proline content significantly decreased, while the concentrations of fructose and G6P returned to CA levels (*p* < 0.05).

Markedly, significantly lower concentrations of amino acids, proline, hexose phosphates (G1P, G6P, and F6P) and glucose (*p* < 0.01), and a significantly higher concentration of sucrose (*p* < 0.01) were observed throughout the entire experiment in crown tissues in treatment II, compared to treatment I (Supplementary Figure 7 and Supplementary Table 10). The crowns accumulated significantly lower concentrations of polyphenols and fructose (*p* < 0.05), and significantly higher concentrations of citrate and starch (*p* < 0.01) on day 7 of treatment II, compared to treatment I. Between days 14 and 21, starch and malate levels increased. As the CA temperature was reduced to 2°C, on days 35–49, the crowns accumulated relatively more F6P, G6P, malate, and sucrose (Figure 5B). Treatment II resulted in lower concentrations of protein, polyphenols and malate (*p* < 0.05), and higher concentrations of CWC, citrate, and starch (*p* < 0.01) after 49 days of CA, in comparison to treatment I (Supplementary Figure 7 and Supplementary Table 10). After DEA (day 56), treatment II was shown to induce lower concentrations of protein content, polyphenols, malate, and fructose (*p* < 0.05), and higher concentrations of CWC and citrate (*p* < 0.05). The levels of starch declined after REA, unlike the concentration of fructose, which remained elevated (Figure 5B and Supplementary Figure 7). Following REA, treatment II was shown to result in significantly lower concentrations of protein content and citrate (*p* < 0.05), and higher concentration of CWC (*p* < 0.001) (Supplementary Figure 7 and Supplementary Table 10).

After DEA, treatment II resulted in significantly increased fructose and starch content, whereas the concentrations of G6P, proline, and sucrose were significantly (*p* < 0.05) lower (Supplementary Table 9). REA resulted in the concentrations of crown starch and sucrose returning to the same levels, as seen after CA in treatment II.

### Correlations of metabolites with winter wheat shoot biomass growth and freezing tolerance

Significant relationships between groups of metabolites, found in leaf and crown tissues, and shoot biomass growth throughout the experiment were determined (Supplementary Figure 8 and Table 1). Treatment I resulted in biomass growth showing strong positive correlations (*r* > 0.6) to chl a, chl b, total chl, protein content, TAC, polyphenols, and sucrose, as well as a moderate (*r* > 0.4) positive correlation to CWC in leaf tissue. Shoot biomass growth likewise correlated with metabolites, found in crown tissue. Thus, a strong positive correlation was observed for sucrose, and moderate positive correlations were found for CWC and starch. Treatment II resulted in very strong (*r* > 0.8) correlations between biomass growth and chl b and polyphenols; strong positive correlations were found to total chl, protein content, CWC, TAC, fructose, and G1P; moderate positive correlations were shown to chl a, malate, G6P, and F6P in leaf tissue (Table 1). Biomass growth also showed strong positive correlations to sucrose and fructose, moderate positive—to CWC and proline, and moderate negative—to protein content and amino acids, measured in crowns (Table 1).

**TABLE 1:** Spearman coefficients (*r*-values) of metabolites against GLA throughout CA, DEA, and REA.

	Treatment I		Treatment II	
	Leaf	Crown	Leaf	Crown
Chl a	0.63	−	0.59	−
Chl b	0.77	−	0.87	−
Total chl	0.76	−	0.73	−
Protein content	0.67	−0.27	0.68	−0.41
CWC	0.43	0.45	0.68	0.57
TAC	0.6	ns	0.76	ns
Polyphenols	0.77	ns	0.89	−0.39
Amino acids	ns	−0.25	ns	−0.51
Proline	ns	ns	ns	0.53
Malate	ns	ns	0.59	ns
Citrate	−	ns	−	−0.26
Starch	0.27	0.48	ns	ns
Sucrose	0.72	0.72	ns	0.77
Glucose	ns	ns	0.36	ns
Fructose	0.34	0.38	0.67	0.79
G1P	ns	ns	0.78	ns
G6P	ns	ns	0.52	ns
F6P	ns	ns	0.5	ns

Very strong (r > 0.8), strong (r > 0.6) and moderate (r > 0.4) positive correlations are indicated by dark, medium and light blue colors. Moderate (r < −0.4) negative correlations are indicated by light red color. Non-significant correlations (p > 0.05) are marked as “ns.” Metabolites, which were not measured in a given tissue, are indicated as “−”.

Furthermore, relationships between leaf and crown tissue metabolites and FT of winter wheat after CA, DEA, and REA were investigated (Supplementary Figure 9). In treatment I, glucose, F6P, G6P, and proline, found in leaves, resulted in statistically significant (*p* < 0.05) positive correlations to FT, ranging from moderate to strong, whereas chl a and total chl showed strong negative correlations (*r* = −0.61; *R* = −0.63), respectively. Proline in crown tissues yielded a very strong positive (*r* = 0.82) and proteins showed a strong positive (*r* = 0.66) correlation with FT in treatment I. In treatment II, proline and proteins showed moderate positive correlations with FT: *r* = 0.59 and *r* = 0.5, respectively. However, both tissues in both treatments showed significant (*p* < 0.05) negative correlations between starch and FT. The strength of these correlations ranged between moderate (*r* = 0.56) and very strong (*r* = 0.83). PCA analyses of metabolites in leaf and crown tissues with LT_30_ as a supplementary factor complement these results (Supplementary Figure 10).

## Discussion

Global climate change may lead to longer and warmer autumns, which will negatively affect the CA capacity of overwintering crops (see reviews by Rapacz et al., 2014; Liu et al., 2019). This poses a problem for future crop production, as insufficient CA can result in freezing damage and, ultimately, significant loss of crop yield. Recent studies have shown that not only CA, but also DEA must be considered in crop breeding—temperature fluctuations in winter can lead to premature DEA, thus, decreasing the FT of overwintering crops (Rapacz et al., 2017, 2022). Moreover, there is a lack of research on the molecular changes, induced by REA. Here, we investigated the metabolite profile dynamics in leaf and crown tissues of 6 winter wheat genotypes with different FT capacity by weekly sampling during the 70-day experiment. Henceforth, the effect of elevated temperature during CA upon shoot biomass growth and FT, and the relationship between these factors and metabolites will be analyzed. Furthermore, the results of this study show consistent differences between metabolite accumulation profiles in source and sink tissues. Finally, the patterns of metabolite accumulation in leaf and crown tissues throughout all stages of acclimation will be discussed.

### Prolonged higher low-temperature during cold acclimation results in increased shoot biomass growth and decreased freezing tolerance

The higher low-temperature treatment during CA resulted in increased shoot biomass accumulation and reduced FT in winter wheat. Twenty-eight days of higher temperature during CA induced irreversibly enhanced shoot biomass accumulation throughout the entire experiment, as compared to constant CA at a lower temperature. Moreover, the growth continued even after the temperature was reduced. Increased biomass production, as well as decreased FT had likewise been recorded in forage grass species (Dalmannsdottir et al., 2017; Jørgensen et al., 2020) and winter wheat (Hanslin and Mortensen, 2010) as a result of elevated temperatures during CA. Jaškūnė et al. (2022) showed similar tendencies of leaf elongation rate and FT in a field experiment among the 6 winter wheat genotypes, chosen for this study. Furthermore, the authors demonstrated a negative relationship between winter wheat leaf growth and FT.

The majority of metabolites in leaf tissues displayed moderate or strong positive correlations with winter wheat shoot biomass accumulation. However, while protein content in leaves had a strong positive relationship, the protein content and amino acids in crowns demonstrated a negative correlation with shoot biomass accumulation, ranging from weak to moderate depending on the treatment. Additionally, a positive relationship was detected between proline and protein content in crowns, and FT. CA leads to decreased levels of carbohydrate anabolism proteins, and increased levels of proteins, which act in stress response, redox metabolism, and carbohydrate catabolism in crowns of winter wheat, thus playing a role in stress tolerance (Kosová et al., 2013). Furthermore, CA had previously been shown to induce the CBF1-activated accumulation of DELLA proteins, which play a role both in repressing plant growth, and increasing FT in *A*. *thaliana* (Achard et al., 2008; Lantzouni et al., 2020).

**FIGURE 6:**
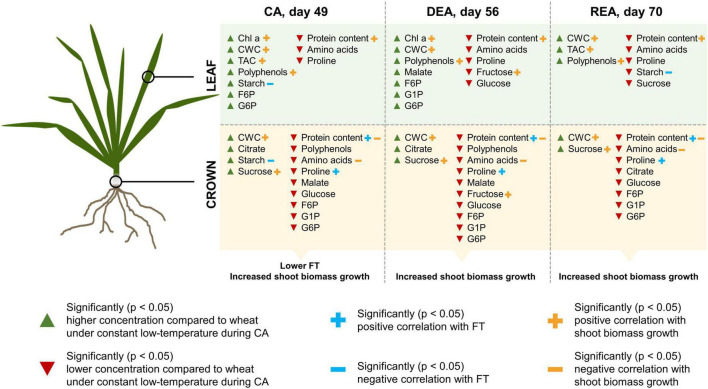
The effect of prolonged higher low-temperature treatment on winter wheat metabolite concentrations and physiology throughout CA, DEA, and REA, as compared to constant low-temperature treatment. Only statistically significant (*p* < 0.05) changes are depicted.

Elevated starch content in both tissues was shown to negatively influence the FT of winter wheat after CA, DEA, and REA. Coincidentally, the prolonged higher low-temperature treatment, which negatively affected FT after CA, also resulted in higher accumulation of starch in both leaves and crowns at this stage of acclimation (Figure 6). Zhao L. et al. (2019) report that transgenic tobacco and pear plants, overexpressing a β-Amylase-encoding gene, displayed improved degradation of starch and accumulation of soluble sugars, and thus, were more FT in comparison to WT plants. Furthermore, the present study revealed that starch was the only metabolite consistently affected by genotype—the concentration of starch in leaf tissues showed a genotype-specific pattern after all three stages of acclimation, whereas in crowns, genotype had an effect after DEA and REA (Supplementary Tables 8, 10). The most freezing-tolerant genotypes, “Sedula DS,” “SW Magnifik,” and “Lakaja DS” often displayed lower concentrations of starch in both tissues and treatment groups, as compared with other genotypes. Coincidentally, a negative relationship was found between starch and proline, and protein content in crowns, which likely contribute to increased FT. Therefore, the ability of crowns to allocate their resources of carbon toward the production of nitrogenous metabolites instead of storage in the form of starch may ultimately result in improved FT. Future work should be directed toward the assessment of activity of enzymes, involved in carbon metabolism, as well as starch synthesis and breakdown under different CA temperature conditions. This would provide further knowledge about the effect of ratio between starch synthesis and breakdown upon FT. Subsequently, leaf starch concentrations during CA must be investigated as a possible indicator of FT. Furthermore, as genotype did not significantly affect the accumulation of proline and protein in a consistent manner, future research may be redirected toward the expression of specific cryoprotective or anti-freeze proteins throughout CA, DEA, and REA.

### Leaf and crown tissues possess distinct metabolite profiles

Higher concentrations of polyphenols, TAC and protein content were identified in leaves under both low-temperature treatment groups at all CA stages, in comparison to the crowns. Abiotic stress induces the production of polyphenols (Šamec et al., 2021). These compounds strengthen the plant cell structure and act as reactive oxygen species (ROS) scavengers, thus, providing protection from freezing injury (Naikoo et al., 2019). This antioxidant activity is further substantiated by polyphenols showing a strong correlation with TAC in leaf tissue throughout the entire experiment (Supplementary Figure 8). Farfan-Vignolo and Asard (2012) similarly describe an increase of polyphenols and TAC in *Lolium perenne* L. and *Medicago sativa* L. under drought stress. Notably, the concentrations of both polyphenols and TAC in leaves showed a constant rise throughout the entire experiment, while the dynamics of these metabolites in the crowns remained relatively stable (Supplementary Figures 3, 4). Photosynthesis generates high amounts of ROS in leaf tissues, and antioxidants are required to maintain the intracellular balance, as well as to alleviate redox stress at low temperatures (Asada, 2006). Furthermore, higher protein content in leaves may be explained by numerous proteins, required for the light and dark reactions of photosynthesis, as well as enzymes involved in carbon or nitrogen metabolism and antioxidant activity (Xu et al., 2013). In this study, increased shoot biomass growth, observed in higher low-temperature conditions, was also reflected in elevated CWC (Supplementary Figure 6). This is supported by moderate and strong positive correlations between leaf CWC and shoot biomass growth, shown in treatments I and II, respectively (Supplementary Figure 8). In contrast, the increased levels of such metabolites as amino acids, proline, hexoses, hexose phosphates, and organic acids in the crowns at all stages of acclimation, in comparison to leaves (Supplementary Tables 5, 6), must be related to the role of this tissue as a meristematic region. The actively dividing shoot apical meristem (SAM) cells within the crown region are vital to cold perception and ensured vernalization of overwintering plants, as well as spring re-growth after the above-ground tissues are lost to freezing damage (Wellensiek, 1964; Tanino and McKersie, 1985). Amino acids and soluble sugars can lower the freezing point and act as cryoprotectants during CA (Bocian et al., 2015; Chai et al., 2019; Livingston et al., 2021). The increased accumulation of hexose phosphates in crowns, as compared to the leaves, indicates the active breakdown of starch or sucrose into glucose and fructose (Kaplan et al., 2007). An increase of hexose phosphates had been previously reported in cold stressed winter wheat (Shahryar and Maali-Amiri, 2016) and they can function as ROS scavengers (Spasojević et al., 2009). Moreover, Xu et al. (2020) propose that increased concentrations of citrate and malate, together with upregulated expression of specific enzyme-coding genes indicate the elevated activity of the citric acid cycle, required for ATP synthesis under stress conditions. Therefore, the increased concentrations of amino acids, hexose phosphates, soluble carbohydrates, and organic acids in the crowns may contribute to the survival of SAM cells by ensuring a stable environment safe from freezing injury, as well as providing energy, required for continuous cell division and eventual regrowth.

### Temperature affects carbon and nitrogen metabolism during cold acclimation in both leaves and crowns

A higher concentration of chl a after 7 and 49 days of CA, as well as higher concentrations of total chl and proteins after 7 days of CA were observed in the leaves of winter wheat under higher low-temperature treatment, in comparison to constant low-temperature treatment. The rate of photosynthesis is highly dependent upon the energy consumption of the plant, and vice versa. Low temperatures are known to induce an energy imbalance between photosynthesis and metabolism by slowing down the rate of the metabolic reactions (Huner et al., 1998). The stress-induced reduction of photosynthetic efficiency is reflected by decreased chlorophyll content (Wang et al., 2016). Nevertheless, winter type crops possess the ability to retain a higher level of photosynthetic activity during CA, compared to spring type crops (Dahal et al., 2012). In comparison to spring cultivars, winter wheat exhibit an increase in sucrose concentrations and sucrose-phosphate synthase activity, ultimately leading to elevated sucrose/starch ratio (Savitch et al., 1997). Starch degradation in photosynthetic tissues plays a significant role in plant stress response (Thalmann and Santelia, 2017), as the resulting soluble sugars invoke osmoprotection and compensate for slowed metabolism (see review by Dong and Beckles, 2019). This is further substantiated by Bocian et al. (2015) and Chai et al. (2019), as low temperature stress in *L. perenne* and *Vitis* species leads to the accumulation of soluble carbohydrates, which act as cryoprotectants.

The prolonged higher low-temperature treatment yielded lower concentrations of hexoses, hexose phosphates, and malate, and a higher concentration of polyphenols in leaves after 7 days of CA, as well as higher concentrations of starch in leaves throughout CA. Hexoses and hexose phosphates are the direct products of starch or sucrose breakdown (Kaplan et al., 2007). Therefore, the first 7 days of CA under two contrasting temperature treatments—prolonged high, and constant low—must have resulted in substantial differences in starch degradation activity. The plants, subjected to warmer conditions experienced a lower level of stress and thus were able to effectively carry out photosynthetic and metabolic processes, as reflected by increased concentrations of chl a, total chl and protein content, resulting in a significantly higher concentration of stored starch. In contrast, the constant low-temperature during CA led to decreased photosynthetic activity and increased breakdown of starch, resulting in the accumulation of soluble carbohydrates and hexose phosphates, which act as cryoprotectants and ROS scavengers. Both treatments resulted in identical leaf sucrose levels during CA, however, the crowns contained higher concentrations of sucrose, while the concentrations of glucose and hexose phosphates had been lower under the higher low-temperature treatment. Therefore, increased photosynthetic activity in higher low-temperature conditions results in an excess of sugars, which are then polymerized for storage (MacNeill et al., 2017).

The accumulation patterns of protein content, amino acids and proline in both leaves and crowns during CA showed an opposite tendency, compared to carbohydrates. Constant low-temperature led to the increased accumulation of these nitrogenous metabolites in comparison with the higher low-temperature treatment. Elevated leaf protein content can help overcome the decreased reaction rates, induced by low temperatures (Strand et al., 1999). Moreover, dehydrins—proteins, which act as protectants against abiotic stress—have been shown to accumulate in winter wheat leaves and crowns during CA, and to positively correlate with FT (Vítámvás et al., 2019, 2021). Elevated amino acid concentrations are likely to contribute to increased FT by acting as osmoregulators (Livingston et al., 2021), which is not the case with starch. These differences between carbon and nitrogen metabolism in the two treatment groups may be the reason behind significantly decreased FT of winter wheat, grown under higher low-temperature CA conditions (Figure 6).

### The ratio between starch accumulation and breakdown during deacclimation and reacclimation influences freezing tolerance

DEA resulted in a significant decrease of FT in the majority of winter wheat genotypes. After 7 days of DEA, the leaves of winter wheat in both treatments showed increased chlorophyll a and starch concentrations, whereas the concentrations of F6P, G6P, glucose, sucrose, and proline had significantly decreased, compared to CA. The crown tissues of DEA plants in both treatments showed a significant increase in fructose and starch, while a decrease of G6P, sucrose and proline were observed. Rys et al. (2020) investigated the effect of DEA on FT and metabolite profiles of winter rapeseed (*Brassica napu*s ssp. *oleifera* L.). The FT of DEA plants was likewise reduced and their leaves contained lower concentrations of soluble sugars and higher water content, in comparison to CA plants. As discussed previously, elevated photosynthetic activity under higher low-temperature conditions can lead to an imbalance between carbon fixation and growth, which may result in increased starch storage. The reduction of sucrose and proline in both tissues may reflect the decreased requirement for cryoprotectant metabolites. Moreover, lower hexose-phosphates levels can be related to increased fluxes in sugar metabolism (Strand et al., 1999). These changes in the metabolite profiles are likely to have led to strongly diminished FT of winter wheat after DEA. While significantly higher levels of starch were observed in leaves and crowns under the higher low-temperature treatment during CA, the concentration of starch in these tissues became similar in both treatments after DEA. Concurrently, our results demonstrated, that at the stage of DEA, there was no difference between the FT of two winter wheat treatment groups. The dynamics of starch accumulation during this stage in both tissues reveal that the concentration of starch began to increase in treatment I, whereas in treatment II, it started to drop. It is possible, that the concentration of starch in both treatment groups returned to a certain level to maintain the balance between photosynthesis and carbon metabolism, thus, resulting in similar FT under both constant low- and prolonged higher low-temperature treatments.

Currently, there is a considerable lack of research on REA and its effect upon the survival of winter type crops, especially wheat. Previous studies have shown that some overwintering species are able to regain their FT after a DEA event. According to Trischuk et al. (2014), REA in winter rapeseed results in fully regained FT, however, winter wheat are not able to fully recover their FT. The authors propose that this is caused by inadequate re-accumulation of soluble carbohydrates in winter wheat. The concentrations of dehydrin proteins increased after REA, thus, suggesting that the interplay of soluble carbohydrates and proteins may regulate FT. However, in the experiment, described by Trischuk et al. (2014), DEA lasted 14 days at 23°C, whereas in the present study, DEA lasted 7 days at 10°C. Our results demonstrate that after REA, leaf tissues again displayed an increase of hexose phosphates, malate, and proline in treatment I, whereas in treatment II, the concentrations of fructose and malate increased and the concentrations of chl a, amino acids decreased, in comparison with DEA. Ultimately, once again starch appears to be central in the response to low-temperature stress. In comparison to DEA, REA resulted in significantly lower leaf starch concentrations, however, this decrease was even more prominent in crown tissues, where starch returned to CA levels in both treatment groups.

In this study, the majority of genotypes had the capacity to regain FT after REA. Metabolite accumulation patterns showed a tendency to repeatedly deplete the starch reserves and to increase the concentrations of soluble carbohydrates, hexose phosphates, and proline. Considering the similar patterns, leading to increased FT after CA, these tendencies in carbon and nitrogen metabolism can be the reason behind regained FT after REA as well. The dissimilarity of our results, in comparison to those, reported by Trischuk et al. (2014), are likely to be caused by the differences of experimental design. Furthermore, the ability of winter wheat to undergo REA can be significantly diminished once full vernalization saturation is reached, which on average takes 49 days (Gusta et al., 1997; Mahfoozi et al., 2001). According to Gorash et al. (2017), the optimum vernalization length of winter wheat can vary from 35 to 63 days. Therefore, genotypes possessing different vernalization saturation requirements may yield varying results of FT following REA. In the future, it is important to investigate the effects of varying DEA temperature and length regimes upon the metabolite profiles and consequent FT of winter type crops after REA.

## Conclusion

The present study provides valuable insight into the physiological and metabolite-level dynamics of six winter wheat genotypes during 3 stages of acclimation under the simulated warmer autumn scenario. The prolonged higher low-temperature treatment significantly increased the accumulation of shoot biomass and had a negative impact on FT of winter wheat. Different patterns of metabolite accumulation in leaf and crown tissues were observed, thus, demonstrating the sink and source interaction, as well as reflecting the photosynthetic and storage functions these two tissues carry out, respectively. The simulated warmer autumn scenario likewise resulted in decreased concentrations of soluble carbohydrates, amino acids, and protein content, as well as increased concentrations of starch in both tissues after CA. DEA negatively affected the FT of winter wheat, decreased the concentrations of G6P, sucrose and proline, and elevated the concentrations of starch in leaves and crowns. This research reveals that winter wheat were able to regain their FT after REA, resulting in increased concentrations of glucose and malate in leaves, higher levels of sucrose in crowns, and decreased concentrations of starch in both tissues. Genotypes of winter wheat played an important role in accumulation of starch in both tissues, and the concentration of this metabolite had a negative relationship with FT throughout all three stages of acclimation. Moreover, starch displayed a negative relationship with proline and proteins in crowns, which coincidentally showed a positive relationship with FT at all 3 stages of acclimation. The balance between starch biosynthesis and breakdown, as well as the ability to direct the products of starch breakdown toward the production of proline and proteins could be key to the survival of winter type crops under changing climate conditions.

## Data availability statement

The original contributions presented in this study are included in the article/Supplementary material, further inquiries can be directed to the corresponding author/s.

## Author contributions

RA conceptualized the idea of the study. RA and GV designed the experiments, acquired the funding, performed the experiments, analyzed the data, visualized the results, and wrote the first draft. YG provided methodology, reagents, and infrastructure for metabolite assays. AA provided advice and help with data analysis. RA, AA, and YG revised and edited the manuscript. All authors contributed to the article and approved the submitted version.

## Footnotes

1
http://digicamcontrol.com
