# Supplementary material for: The effect of cold acclimation, deacclimation and reacclimation on metabolite profiles and freezing tolerance in winter wheat
Source: Front Plant Sci. 2022 Aug 15;13:959118. doi: 10.3389/fpls.2022.959118 (PMC9421140; doi:10.3389/fpls.2022.959118)
Supplement: Supplementary file 1 [file Data_Sheet_1.docx]

Supplementary Material

**Supplementary Table 1.** Descriptive statistics and Wilcoxon rank sum test results of GLA between treatments at each time-point.

| **Day** | **Treatment I**  **Mean ± SD** | **Treatment II**  **Mean ± SD** | **p-value** |
| --- | --- | --- | --- |
| 0 | 9.57 ± 1.24 | 9.91 ± 1.90 | 0.4064 |
| 7 | 10.36 ± 1.61 | 15.00 ± 2.86 | 1.111e-05 *** |
| 14 | 11.55 ± 1.79 | 25.43 ± 5.10 | 2.204e-10 *** |
| 21 | 12.69 ± 1.96 | 42.70 ± 8.00 | 2.204e-10 *** |
| 28 | 14.57 ± 2.54 | 66.53 ± 12.69 | 4.408e-10 *** |
| 35 | 17.78 ± 3.50 | 81.18 ± 16.82 | 2.204e-10 *** |
| 42 | 22.37 ± 5.37 | 94.29 ± 18.92 | 2.204e-10 *** |
| 49 | 24.78 ± 6.32 | 103.38 ± 20.43 | 2.204e-10 *** |
| 56 | 36.55 ± 8.08 | 127.32 ± 24.26 | 2.204e-10 *** |
| 63 | 48.29 ± 12.02 | 147.59 ± 28.86 | 2.204e-10 *** |
| 70 | 54.19 ± 13.46 | 150.26 ± 29.27 | 2.204e-10 *** |

**** indicates significant differences at p < 0.001.*

**Supplementary Table 2.** Descriptive statistics and ANOVA results of the effect of genotype on GLA of winter wheat in treatments I and II during CA, DEA and REA.

| **Treatment** | **Stage** | **Genotype** | **Mean ± SD** | **D.f.** | **Sum of Squares** | **Mean of Squares** | **F value** | **p-value** | ***Post hoc* Tukey’s HSD** |
| --- | --- | --- | --- | --- | --- | --- | --- | --- | --- |
| I | CA, day 49 | KWS Ferrum | 28.44 ± 2.77 | 5 | 588.3 | 117.67 | 15.47 | 7.17e-05 *** | ab |
|  |  | Hanswin | 33.94 ± 2.20 |  |  |  |  |  | a |
|  |  | Nordkap | 24.17 ± 1.88 |  |  |  |  |  | bc |
|  |  | Sedula DS | 17.91 ± 2.05 |  |  |  |  |  | c |
|  |  | SW Magnifik | 26.42 ± 4.60 |  |  |  |  |  | ab |
|  |  | Lakaja DS | 17.81 ± 2.07 |  |  |  |  |  | c |
|  | DEA, day 56 | KWS Ferrum | 41.63 ± 6.28 | 5 | 858.0 | 171.60 | 8.214 | 0.00142 ** | a |
|  |  | Hanswin | 46.98 ± 3.14 |  |  |  |  |  | a |
|  |  | Nordkap | 35.24 ± 2.71 |  |  |  |  |  | ab |
|  |  | Sedula DS | 28.41 ± 2.52 |  |  |  |  |  | b |
|  |  | SW Magnifik | 39.21 ± 7.57 |  |  |  |  |  | ab |
|  |  | Lakaja DS | 27.81 ± 2.23 |  |  |  |  |  | b |
|  | REA, day 70 | KWS Ferrum | 67.06 ± 10.30 | 5 | 2501.0 | 500.2 | 10.36 | 5e-04 *** | a |
|  |  | Hanswin | 68.93 ± 7.59 |  |  |  |  |  | a |
|  |  | Nordkap | 52.76 ± 7.08 |  |  |  |  |  | abc |
|  |  | Sedula DS | 41.00 ± 3.31 |  |  |  |  |  | bc |
|  |  | SW Magnifik | 57.47 ± 7.64 |  |  |  |  |  | ab |
|  |  | Lakaja DS | 37.94 ± 2.54 |  |  |  |  |  | c |
| II | CA, day 49 | KWS Ferrum | 106.50 ± 13.05 | 5 | 5451 | 1090.2 | 7.94 | 0.00164 ** | abc |
|  |  | Hanswin | 127.84 ± 5.69 |  |  |  |  |  | a |
|  |  | Nordkap | 99.29 ± 19.48 |  |  |  |  |  | abc |
|  |  | Sedula DS | 75.87 ± 13.38 |  |  |  |  |  | c |
|  |  | SW Magnifik | 119.99 ± 6.97 |  |  |  |  |  | ab |
|  |  | Lakaja DS | 90.77 ± 3.71 |  |  |  |  |  | bc |
|  | DEA, day 56 | KWS Ferrum | 135.53 ± 12.01 | 5 | 8500 | 1700.0 | 13.57 | 0.000138 *** | ab |
|  |  | Hanswin | 153.01 ± 7.58 |  |  |  |  |  | a |
|  |  | Nordkap | 123.84 ± 19.84 |  |  |  |  |  | ab |
|  |  | Sedula DS | 91.85 ± 6.47 |  |  |  |  |  | c |
|  |  | SW Magnifik | 150.13 ± 10.54 |  |  |  |  |  | a |
|  |  | Lakaja DS | 109.57 ± 1.88 |  |  |  |  |  | bc |
|  | REA, day 70 | KWS Ferrum | 163.92 ± 20.56 | 5 | 12414 | 2482.8 | 13.86 | 0.000124 *** | ab |
|  |  | Hanswin | 183.66 ± 11.07 |  |  |  |  |  | a |
|  |  | Nordkap | 141.42 ± 17.77 |  |  |  |  |  | bc |
|  |  | Sedula DS | 110.73 ± 11.79 |  |  |  |  |  | c |
|  |  | SW Magnifik | 175.33 ± 3.58 |  |  |  |  |  | ab |
|  |  | Lakaja DS | 126.46 ± 7.88 |  |  |  |  |  | c |

*** indicates significant differences at p < 0.01, and *** at p < 0.001. Post hoc Tukey’s HSD results are depicted as letters, where different letters indicate statistically significant (p < 0.05) differences between means.*

**Supplementary Table 3.** Two-way ANOVA and Kruskal Wallis test results of the effect of genotype, treatment and interaction between genotype and treatment on LT_30_ values of winter wheat. Kruskal Wallis test was applied to DEA data, as it did not meet the requirements of ANOVA.

| **Stage** | **Source** | **D.f.** | **Sum of squares** | **Mean of squares** | **F value** | ***X*^2^** | **p-value** |
| --- | --- | --- | --- | --- | --- | --- | --- |
| CA, day 56 | Genotype | 5 | 158.10 | 31.619 | 22.899 | - | 4.23e-09 *** |
|  | Treatment | 1 | 12.87 | 12.866 | 9.318 | - | 0.00493 ** |
|  | Interaction | 5 | 10.11 | 2.021 | 1.464 | - | 0.23306 |
| DEA, day 56 | Genotype | 5 | - | - | - | 27.124 | 5.395e-05 *** |
|  | Treatment | 1 | - | - | - | 1.3779 | 0.2405 |
|  | Interaction | - | - | - | - | - | - |
| REA, day 70 | Genotype | 5 | 121.35 | 24.270 | 28.826 | - | 2.58e-11 *** |
|  | Treatment | 1 | 1.20 | 1.198 | 1.423 | - | 0.241 |
|  | Interaction | 5 | 4.46 | 0.893 | 1.060 | - | 0.399 |

*** indicates significant differences at p < 0.01, and *** at p < 0.001.*


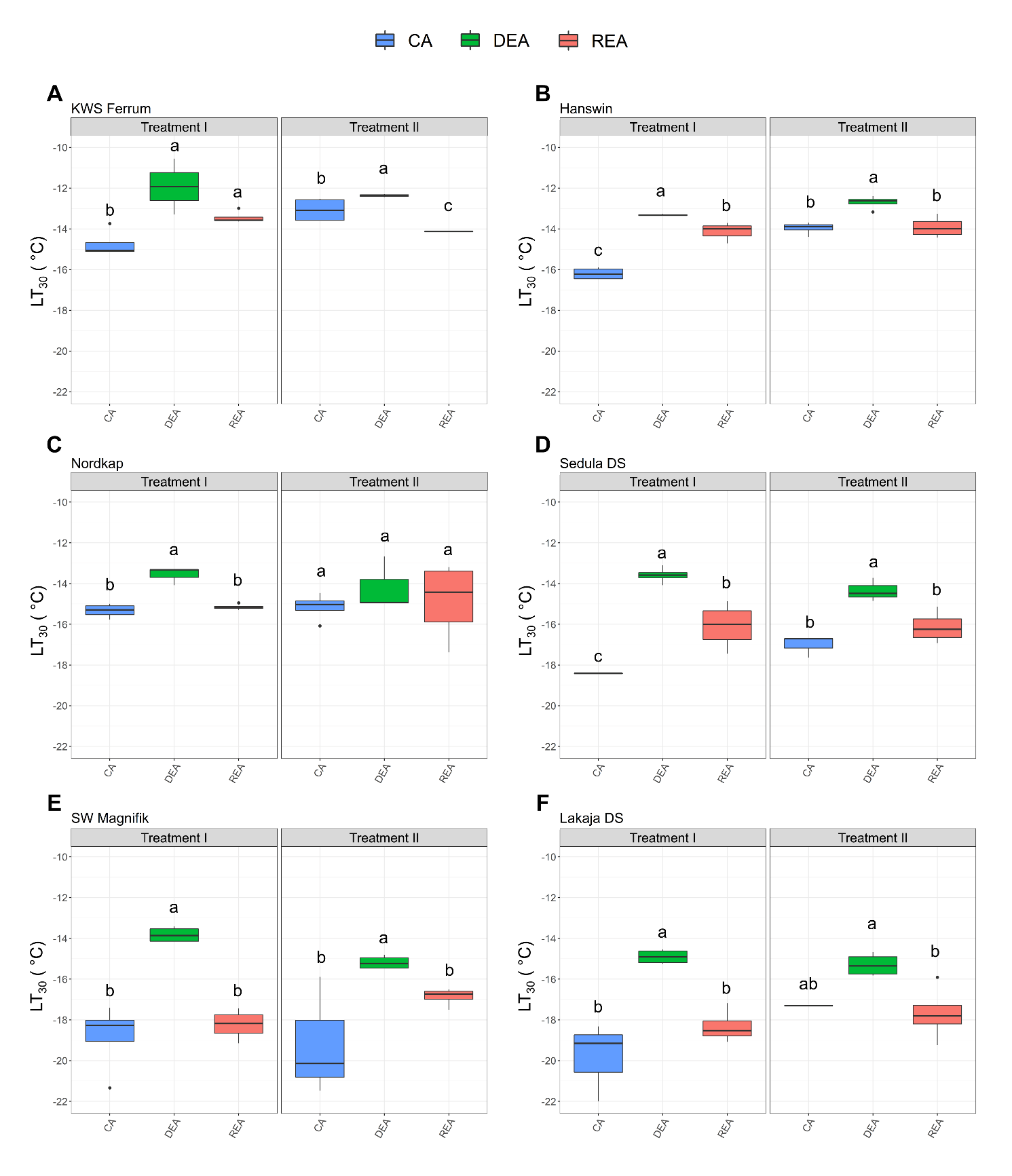


**Supplementary Figure 1.** Differences in FT of ‘KWS Ferrum’ (**A**), ‘Hanswin’ (**B**), ‘Nordkap’ (**C**), ‘Sedula DS’ (**D**), ‘SW Magnifik’ (**E**) and ‘Lakaja DS’ (**F**) between the three stages of acclimation. Letters above the boxplots signify statistically significant (p < 0.05) differences between stages of acclimation within each treatment group.

**Supplementary Table 4.** Descriptive statistics and ANOVA/Kruskal Wallis test results of the effect of genotype on LT_30_ values of winter wheat in treatments I and II during CA, DEA and REA. Kruskal Wallis test was applied to DEA data, as it did not meet the requirements of ANOVA.

| **Treatment** | **Stage** | **Genotype** | **Mean ± SD** | **D.f.** | **Sum of Squares** | **Mean of Squares** | **F value** | ***X*^2^** | **p-value** | ***Post hoc* Tukey’s HSD** |
| --- | --- | --- | --- | --- | --- | --- | --- | --- | --- | --- |
| I | CA, day 49 | KWS Ferrum | -14.74 **±** 0.67 | 5 | 75.17 | 15.035 | 12.3 | - | 7.22e-05 *** | a |
|  |  | Hanswin | -16.20 **±** 0.30 |  |  |  |  |  |  | ab |
|  |  | Nordkap | -15.33 **±** 0.34 |  |  |  |  |  |  | ab |
|  |  | Sedula DS | -18.41 **±** NA |  |  |  |  |  |  | bc |
|  |  | SW Magnifik | -18.82 **±** 1.73 |  |  |  |  |  |  | c |
|  |  | Lakaja DS | -19.82 **±** 1.92 |  |  |  |  |  |  | c |
|  | DEA, day 56 | KWS Ferrum | -11.93 **±** 1.94 | 5 | - | - | - | 14.391 | 0.01331 * | a |
|  |  | Hanswin | -13.32 **±** 0.04 |  |  |  |  |  |  | ab |
|  |  | Nordkap | -13.57 **±** 0.44 |  |  |  |  |  |  | abc |
|  |  | Sedula DS | -13.59 **±** 0.40 |  |  |  |  |  |  | bc |
|  |  | SW Magnifik | -13.82 **±** 0.38 |  |  |  |  |  |  | c |
|  |  | Lakaja DS | -14.91 **±** 0.36 |  |  |  |  |  |  | d |
|  | REA, day 70 | KWS Ferrum | -13.45 **±** 0.30 | 5 | 81.04 | 16.208 | 32.59 | - | 3.91e-08 *** | a |
|  |  | Hanswin | -14.14 **±** 0.51 |  |  |  |  |  |  | ab |
|  |  | Nordkap | -15.15 **±** 0.14 |  |  |  |  |  |  | bc |
|  |  | Sedula DS | -16.08 **±** 1.14 |  |  |  |  |  |  | c |
|  |  | SW Magnifik | -18.24 **±** 0.75 |  |  |  |  |  |  | d |
|  |  | Lakaja DS | -18.33 **±** 0.82 |  |  |  |  |  |  | d |
| II | CA, day 49 | KWS Ferrum | -13.07 **±** 0.60 | 5 | 84.0 | 16.812 | 10.75 | - | 0.000296 *** | a |
|  |  | Hanswin | -13.97 **±** 0.31 |  |  |  |  |  |  | ab |
|  |  | Nordkap | -15.15 **±** 0.68 |  |  |  |  |  |  | ab |
|  |  | Sedula DS | -17.01 **±** 0.53 |  |  |  |  |  |  | bc |
|  |  | SW Magnifik | -19.17 **±** 2.92 |  |  |  |  |  |  | c |
|  |  | Lakaja DS | -17.3 **±** NA |  |  |  |  |  |  | bc |
|  | DEA, day 56 | KWS Ferrum | -12.37 **±** 0.11 | 5 | - | - | - | 14.638 | 0.01203 * | a |
|  |  | Hanswin | -12.70 **±** 0.33 |  |  |  |  |  |  | ab |
|  |  | Nordkap | -14.18 **±** 1.31 |  |  |  |  |  |  | cd |
|  |  | Sedula DS | -14.35 **±** 0.58 |  |  |  |  |  |  | bc |
|  |  | SW Magnifik | -15.19 **±** 0.34 |  |  |  |  |  |  | d |
|  |  | Lakaja DS | -15.30 **±** 0.56 |  |  |  |  |  |  | d |
|  | REA, day 70 | KWS Ferrum | -14.14 **±** 0.02 | 5 | 44.87 | 8.974 | 7.562 | - | 0.000671 *** | a |
|  |  | Hanswin | -13.92 **±** 0.52 |  |  |  |  |  |  | a |
|  |  | Nordkap | -14.85 **±** 1.95 |  |  |  |  |  |  | ab |
|  |  | Sedula DS | -16.14 **±** 0.78 |  |  |  |  |  |  | abc |
|  |  | SW Magnifik | -16.87 **±** 0.44 |  |  |  |  |  |  | bc |
|  |  | Lakaja DS | -17.69 **±** 1.36 |  |  |  |  |  |  | c |

** indicates significant differences at p < 0.05, ** at p < 0.01, and *** at p < 0.001. Post hoc Tukey’s HSD results are depicted as letters, where different letters indicate statistically significant (p < 0.05) differences between means.*


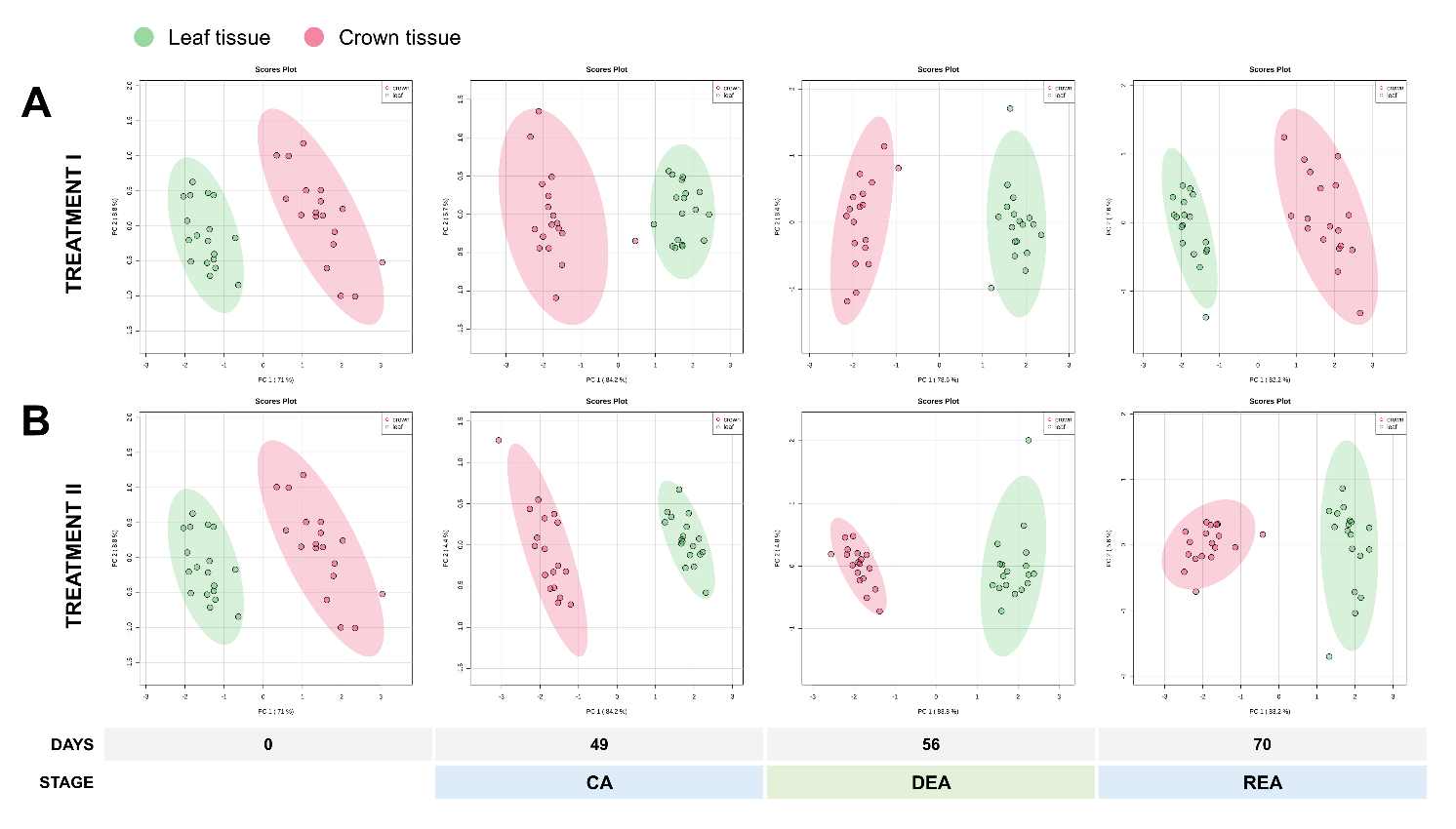


**Supplementary Figure 2.** Differentiation of metabolite profiles between tissues. Score plots of principal component analyses (PCAs), performed with 14 different metabolites in leaf and crown region tissue samples of 6 winter wheat genotypes, measured on day 0 (pre-CA), day 49 (CA), day 56 (DEA), and day 70 (REA) in treatment I (**A**) and treatment II (**B**). Three biological replicates were used for the metabolic analyses. The colored ellipses indicate the 95% confidence limits.


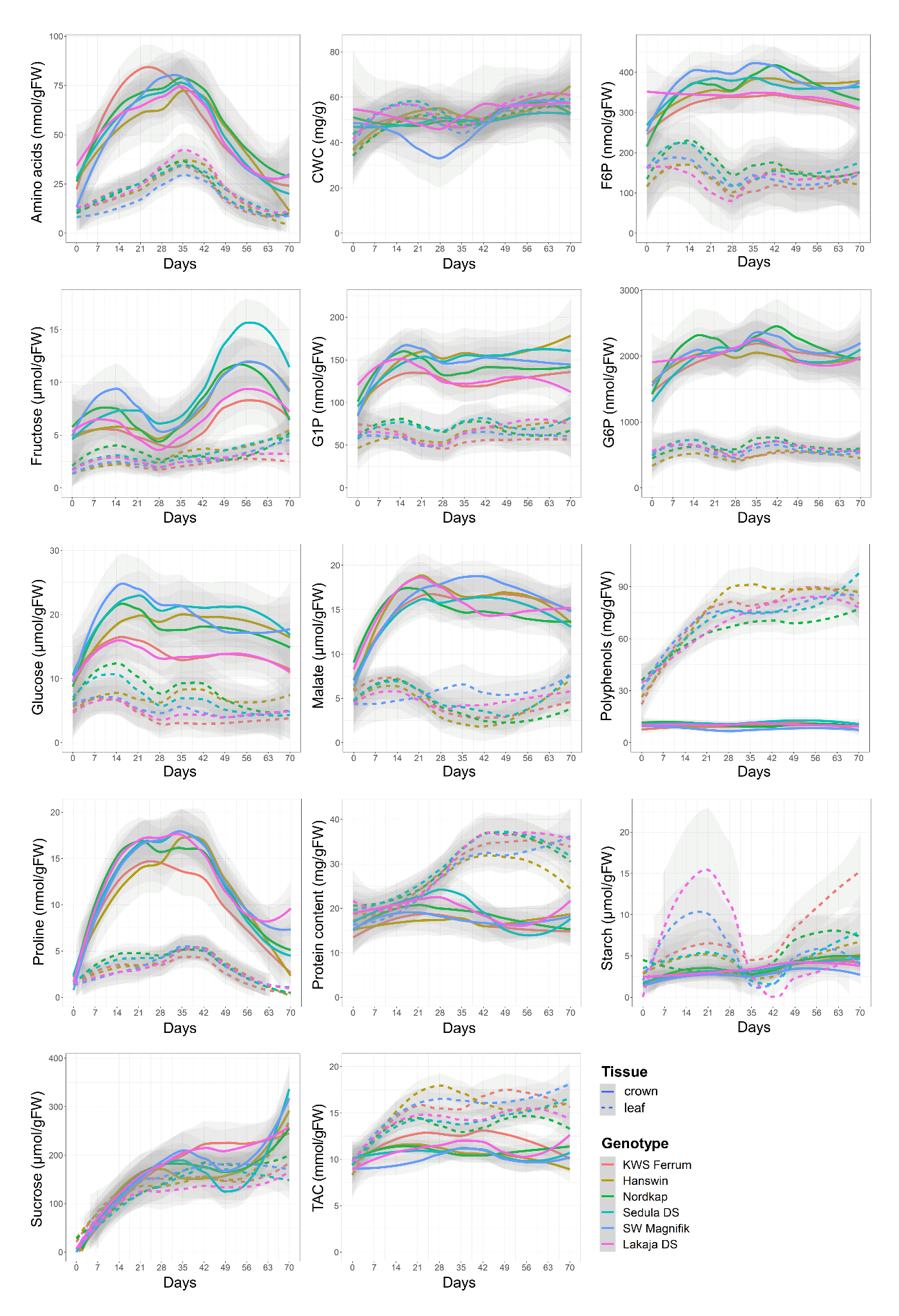
 **Supplementary Figure 3.** Differences in dynamics of 14 different metabolites in leaf and crown tissues of 6 winter wheat genotypes in treatment I during CA, DEA and REA. LOESS regression analysis was applied. The grey areas indicate the 95% confidence interval.

**Supplementary Table 5.** Descriptive statistics and Wilcoxon rank sum test results of metabolite concentration values between tissues in treatment I.

|  | **NA, day 0** | | | **CA, day 49** | | | **DEA, day 56** | | | **REA, day 70** | | |
| --- | --- | --- | --- | --- | --- | --- | --- | --- | --- | --- | --- | --- |
| **Day** | **Leaf tissue**  **Mean ± SD** | **Crown tissue**  **Mean ± SD** | **p-value** | **Leaf tissue**  **Mean ± SD** | **Crown tissue**  **Mean ± SD** | **p-value** | **Leaf tissue**  **Mean ± SD** | **Crown tissue**  **Mean ± SD** | **p-value** | **Leaf tissue**  **Mean ± SD** | **Crown tissue**  **Mean ± SD** | **p-value** |
| Protein content (mg/gFW) | 18.83 ± 2.88 | 16.10 ± 3.58 | 0.0266 * | 35.96 ± 5.50 | 16.60 ± 3.70 | 4.408e-10 *** | 32.52 ± 4.79 | 14.27 ± 3.54 | 2.204e-10 *** | 31.58 ± 6.35 | 16.74 ± 6.05 | 4.595e-07 *** |
| CWC (mg/g) | 40.48 ± 10.25 | 43.46 ± 10.01 | 0.4812 | 54.28 ± 3.95 | 54.16 ± 6.28 | 0.8301 | 57.33 ± 3.83 | 59.22 ± 9.65 | 0.673 | 56.69 ± 5.20 | 57.99 ± 7.29 | 0.7637 |
| TAC (nmol/gFW) | 9.30 ± 1.00 | 9.24 ± 1.05 | 0.3506 | 15.70 ± 2.42 | 10.98 ± 1.63 | 1.322e-08 *** | 15.93 ± 1.72 | 10.44 ± 2.00 | 1.543e-09 *** | 15.73 ± 3.14 | 11.03 ± 3.14 | 3.642e-05 *** |
| Polyphenols (mg/gFW) | 31.90 ± 4.42 | 8.60 ± 2.90 | 2.204e-10 *** | 80.88 ± 10.44 | 10.45 ± 2.68 | 4.813e-07 *** | 87.55 ± 16.10 | 11.31 ± 3.04 | 2.204e-10 *** | 84.32 ± 13.98 | 8.63 ± 2.19 | 2.204e-10 *** |
| Amino acids (nmol/gFW) | 6.47 ± 0.83 | 18.39 ± 5.46 | 2.204e-10 *** | 18.62 ± 5.96 | 46.38 ± 16.91 | 1.199e-07 *** | 11.90 ± 7.27 | 25.68 ± 10.66 | 4.177e-05 *** | 9.28 ± 3.37 | 24.88 ± 12.03 | 9.917e-09 *** |
| Proline (nmol/gFW) | 0.25 ± 0.04 | 0.16 ± 0.07 | 0.0002807 *** | 4.40 ± 1.95 | 14.14 ± 2.34 | 4.408e-10 *** | 0.50 ± 0.26 | 3.38 ± 2.05 | 3.788e-07 *** | 1.05 ± 0.73 | 6.36 ± 3.30 | 3.808e-07 *** |
| Malate (µmol/gFW) | 3.12 ± 1.01 | 6.21 ± 2.42 | 4.892e-05 *** | 3.84 ± 1.71 | 16.60 ± 2.47 | 7.04e-07 *** | 2.87 ± 1.61 | 14.81 ± 2.39 | 4.242e-09 *** | 5.62 ± 2.04 | 14.13 ± 2.11 | 4.408e-10 *** |
| Starch (µmol/gFW) | 4.45 ± 1.80 | 1.58 ± 0.68 | 1.092e-05 *** | 3.49 ± 1.96 | 3.17 ± 0.71 | 0.8045 | 10.32 ± 4.15 | 6.35 ± 1.60 | 0.001122 ** | 8.21 ± 5.31 | 4.62 ± 1.65 | 0.01063 * |
| Sucrose (µmol/gFW) | 6.66 ± 3.55 | 7.29 ± 4.44 | 0.8562 | 180.40 ± 35.63 | 206.14 ± 73.51 | 0.4235 | 132.64 ± 38.98 | 138.46 ± 67.70 | 0.5418 | 201.53 ± 89.65 | 291.54 ± 66.49 | 2.871e-05 *** |
| Glucose (µmol/gFW) | 1.44 ± 0.85 | 4.35 ± 1.59 | 2.611e-06 *** | 5.46 ± 3.13 | 17.96 ± 3.75 | 3.085e-09 *** | 2.92 ± 1.35 | 15.74 ± 3.59 | 2.204e-10 *** | 4.54 ± 2.00 | 13.74 ± 3.32 | 4.187e-09 *** |
| Fructose (µmol/gFW) | 0.38 ± 0.15 | 1.41 ± 0.75 | 3.543e-06 *** | 3.03 ± 1.02 | 8.63 ± 2.93 | 8.74e-07 *** | 3.04 ± 1.43 | 14.39 ± 3.57 | 2.204e-10 *** | 4.09 ± 1.95 | 7.16 ± 2.11 | 6.884e-05 *** |
| G1P (nmol/gFW) | 36.85 ± 12.26 | 72.93 ± 32.96 | 0.0002105 *** | 69.84 ± 18.705 | 147.90 ± 23.64 | 8.815e-10 *** | 61.52 ± 17.29 | 133.81 ± 16.08 | 2.204e-10 *** | 68.49 ± 17.29 | 141.99 ± 23.63 | 4.408e-10 *** |
| G6P (nmol/gFW) | 207.23 ± 64.91 | 1270.80 ± 412.93 | 2.204e-10 *** | 648.28 ± 184.43 | 2084.92 ±  393.96 | 4.408e-10 *** | 432.39 ± 103.24 | 1650.62 ± 194.29 | 2.204e-10 *** | 552.79 ± 101.84 | 1982.31 ± 358.22 | 2.204e-10 *** |
| F6P (nmol/gFW) | 63.70 ± 19.39 | 222.02 ± 88.86 | 2.645e-09 *** | 162.19 ± 72.60 | 386.09 ± 56.24 | 3.085e-09 *** | 75.85 ± 40.72 | 319.88 ± 39.58 | 2.204e-10 *** | 145.66 ± 30.44 | 347.16 ± 47.89 | 2.204e-10 *** |

** indicates significant differences at p < 0.05, ** at p < 0.01, and *** at p < 0.001.*


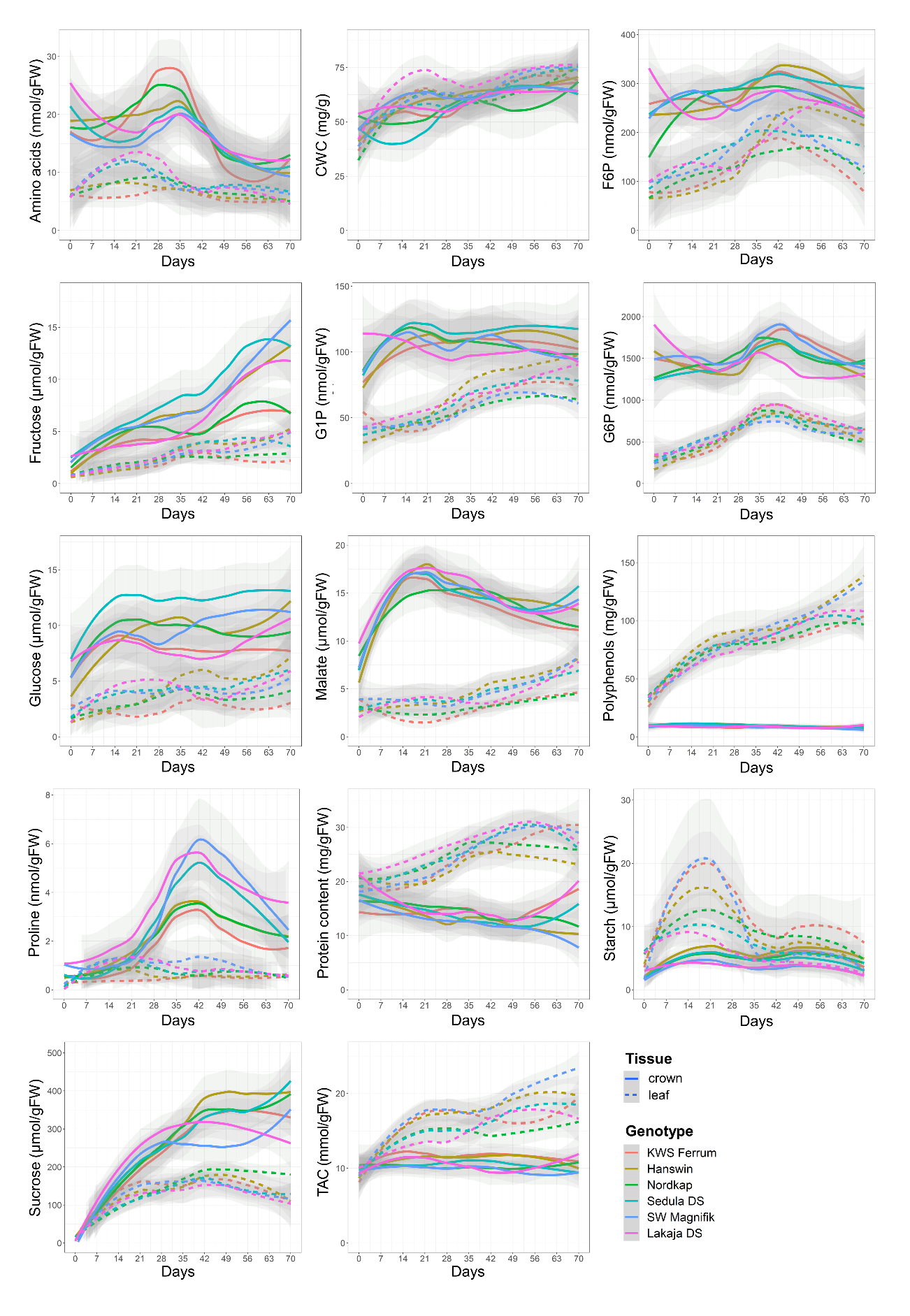


**Supplementary Figure 4.** Differences in dynamics of 14 different metabolites in leaf and crown tissues of 6 winter wheat genotypes in treatment II during CA, DEA and REA. LOESS regression analysis was applied. The grey areas indicate the 95% confidence interval.

**Supplementary Table 6.** Descriptive statistics and Wilcoxon rank sum test results of metabolite concentration values between tissues in treatment II.

|  | **NA, day 0** | | | **CA, day 49** | | | **DEA, day 56** | | | **REA, day 70** | | |
| --- | --- | --- | --- | --- | --- | --- | --- | --- | --- | --- | --- | --- |
| **Day** | **Leaf tissue**  **Mean ± SD** | **Crown tissue**  **Mean ± SD** | **p-value** | **Leaf tissue**  **Mean ± SD** | **Crown tissue**  **Mean ± SD** | **p-value** | **Leaf tissue**  **Mean ± SD** | **Crown tissue**  **Mean ± SD** | **p-value** | **Leaf tissue**  **Mean ± SD** | **Crown tissue**  **Mean ± SD** | **p-value** |
| Protein content (mg/gFW) | 18.83 ± 2.88 | 16.10 ± 3.58 | 0.0266 * | 29.47 ± 4.79 | 13.06 ± 2.77 | 3.222e-07 *** | 28.69 ± 4.30 | 11.55 ± 2.54 | 3.222e-07 *** | 26.75 ± 3.67 | 13.33 ± 7.67 | 1.64e-05 *** |
| CWC (mg/g) | 40.48 ± 10.25 | 43.46 ± 10.01 | 0.4812 | 69.82 ± 5.71 | 65.11 ± 4.82 | 0.01086 * | 72.24 ± 5.89 | 63.71 ± 12.97 | 0.00379 ** | 73.85 ± 5.01 | 67.33 ± 3.75 | 0.0002914 *** |
| TAC (nmol/gFW) | 9.30 ± 1.00 | 9.24 ± 1.05 | 0.3506 | 18.17 ± 3.49 | 10.88 ± 1.75 | 1.12e-07 *** | 17.21 ± 3.54 | 9.89 ± 1.95 | 6.611e-09 *** | 18.96 ± 3.15 | 10.36 ± 1.89 | 1.543e-09 *** |
| Polyphenols (mg/gFW) | 31.90 ± 4.42 | 8.60 ± 2.90 | 2.204e-10 *** | 99.68 ± 12.93 | 8.42 ± 1.76 | 2.204e-10 *** | 100.65 ± 18.74 | 7.38 ± 2.14 | 2.204e-10 *** | 110.26 ± 31.59 | 8.27 ± 2.60 | 2.204e-10 *** |
| Amino acids (nmol/gFW) | 6.47 ± 0.83 | 18.39 ± 5.46 | 2.204e-10 *** | 7.37 ± 1.78 | 12.71 ± 3.90 | 7.696e-07 *** | 6.60 ± 1.99 | 10.11 ± 3.58 | 0.001182 ** | 5.57 ± 1.33 | 10.59 ± 1.97 | 7.343e-07 *** |
| Proline (nmol/gFW) | 0.25 ± 0.04 | 0.16 ± 0.07 | 0.0002807 *** | 0.84 ± 0.44 | 4.71 ± 2.22 | 8.815e-10 *** | 0.49 ± 0.22 | 1.65 ± 0.70 | 2.064e-06 *** | 0.61 ± 0.28 | 2.55 ± 0.85 | 3.2e-07 *** |
| Malate (µmol/gFW) | 3.12 ± 1.01 | 6.21 ± 2.42 | 4.892e-05 *** | 5.21 ± 1.75 | 12.05 ± 2.16 | 3.63e-09 *** | 4.07 ± 1.32 | 12.45 ± 2.07 | 4.805e-07 *** | 6.72 ± 1.91 | 12.99 ± 3.14 | 1.12e-07 *** |
| Starch (µmol/gFW) | 4.45 ± 1.80 | 1.58 ± 0.68 | 1.092e-05 *** | 5.35 ± 2.08 | 4.65 ± 1.84 | 0.4197 | 11.95 ± 4.30 | 7.16 ± 1.94 | 0.0002105 *** | 4.34 ± 2.38 | 3.73 ± 1.18 | 0.8618 |
| Sucrose (µmol/gFW) | 6.66 ± 3.55 | 7.29 ± 4.44 | 0.8562 | 177.76 ± 25.64 | 368.41 ± 106.69 | 2.204e-10 *** | 137.50 ± 30.70 | 295.20 ± 56.05 | 2.204e-10 *** | 135.83 ± 33.71 | 384.35 ± 88.53 | 2.204e-10 *** |
| Glucose (µmol/gFW) | 1.44 ± 0.85 | 4.35 ± 1.59 | 2.611e-06 *** | 4.04 ± 1.60 | 9.78 ± 3.42 | 2.533e-06 *** | 1.78 ± 0.83 | 8.48 ± 3.02 | 6.237e-07 *** | 4.50 ± 2.07 | 9.97 ± 2.72 | 8.768e-06 *** |
| Fructose (µmol/gFW) | 0.38 ± 0.15 | 1.41 ± 0.75 | 3.543e-06 *** | 3.37 ± 1.18 | 7.00 ± 3.08 | 6.884e-05 *** | 2.19 ± 1.58 | 10.66 ± 3.66 | 1.543e-09 *** | 3.61 ± 1.75 | 9.68 ± 3.86 | 6.511e-06 *** |
| G1P (nmol/gFW) | 36.85 ± 12.26 | 72.93 ± 32.96 | 0.0002105 *** | 75.57 ± 9.53 | 108.46 ± 19.89 | 7.696e-07 *** | 71.38 ± 15.63 | 102.33 ± 16.74 | 9.873e-07 *** | 74.25 ± 16.10 | 99.55 ± 20.22 | 0.0009996 *** |
| G6P (nmol/gFW) | 207.23 ± 64.91 | 1270.80 ± 412.93 | 2.204e-10 *** | 781.23 ± 104.30 | 1590.17 ± 367.26 | 8.22e-08 *** | 541.93 ± 111.66 | 1310.20 ± 302.62 | 2.204e-10 *** | 578.38 ± 132.27 | 1387.81 ± 275.93 | 1.543e-09 *** |
| F6P (nmol/gFW) | 63.70 ± 19.39 | 222.02 ± 88.86 | 2.645e-09 *** | 227.69 ± 80.39 | 304.96 ± 56.17 | 0.001849 ** | 140.25 ± 65.76 | 270.23 ± 45.04 | 4.297e-08 *** | 153.32 ± 85.59 | 250.15 ± 40.01 | 4.892e-05 *** |

** indicates significant differences at p < 0.05, ** at p < 0.01, and *** at p < 0.001.*


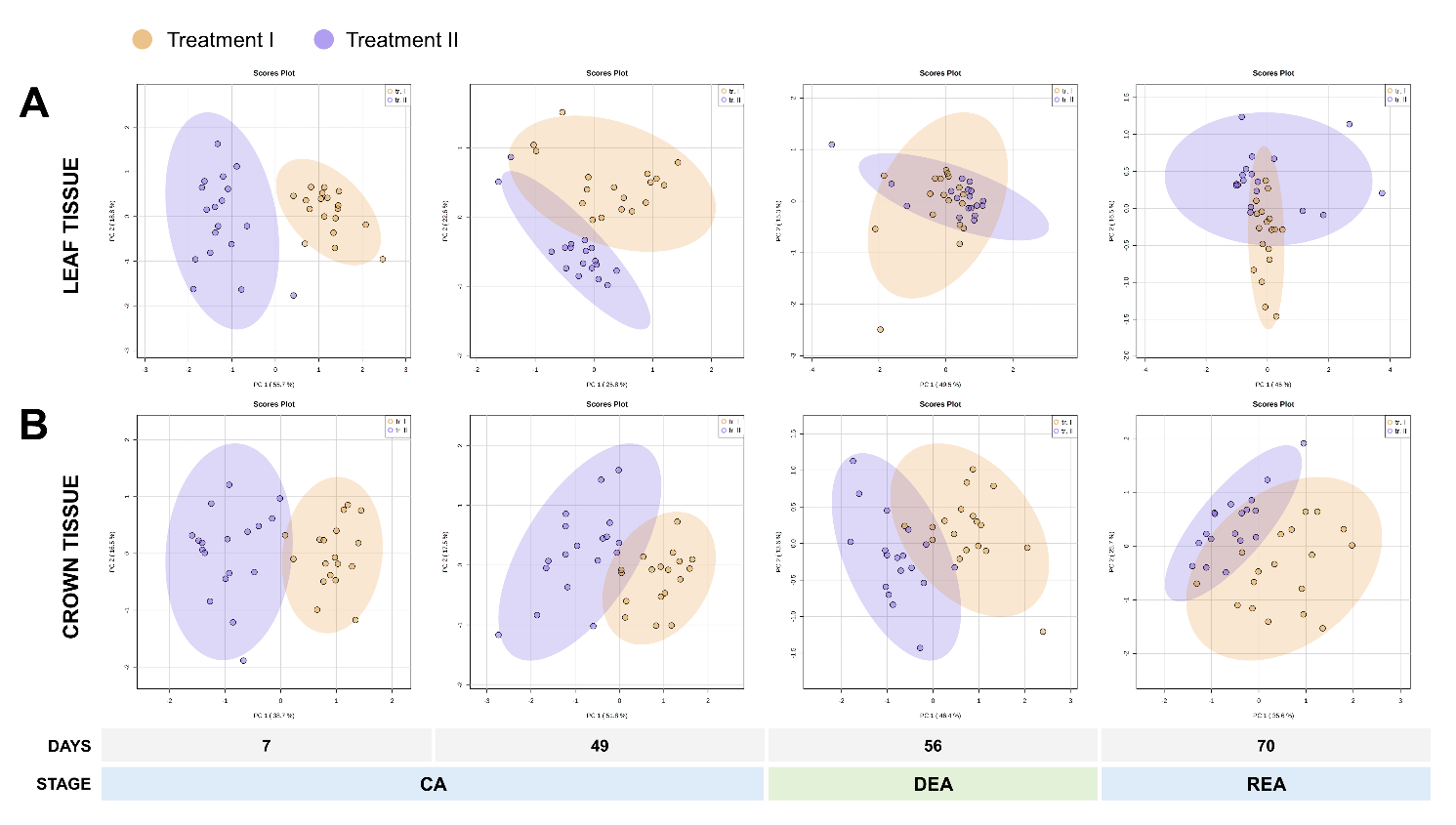


**Supplementary Figure 5.** Differentiation of metabolite profiles between treatments. Score plots of principal component analyses (PCAs), performed with 16 different metabolites in leaves (**A**) and 15 different metabolic traits in crowns (**B**) of 6 winter wheat genotypes, measured on days 7 and 49 (CA), day 56 (DEA), and day 70 (REA) in two treatment groups. Three biological replicates were used for the metabolic analyses. The colored ellipses indicate the 95% confidence limits.


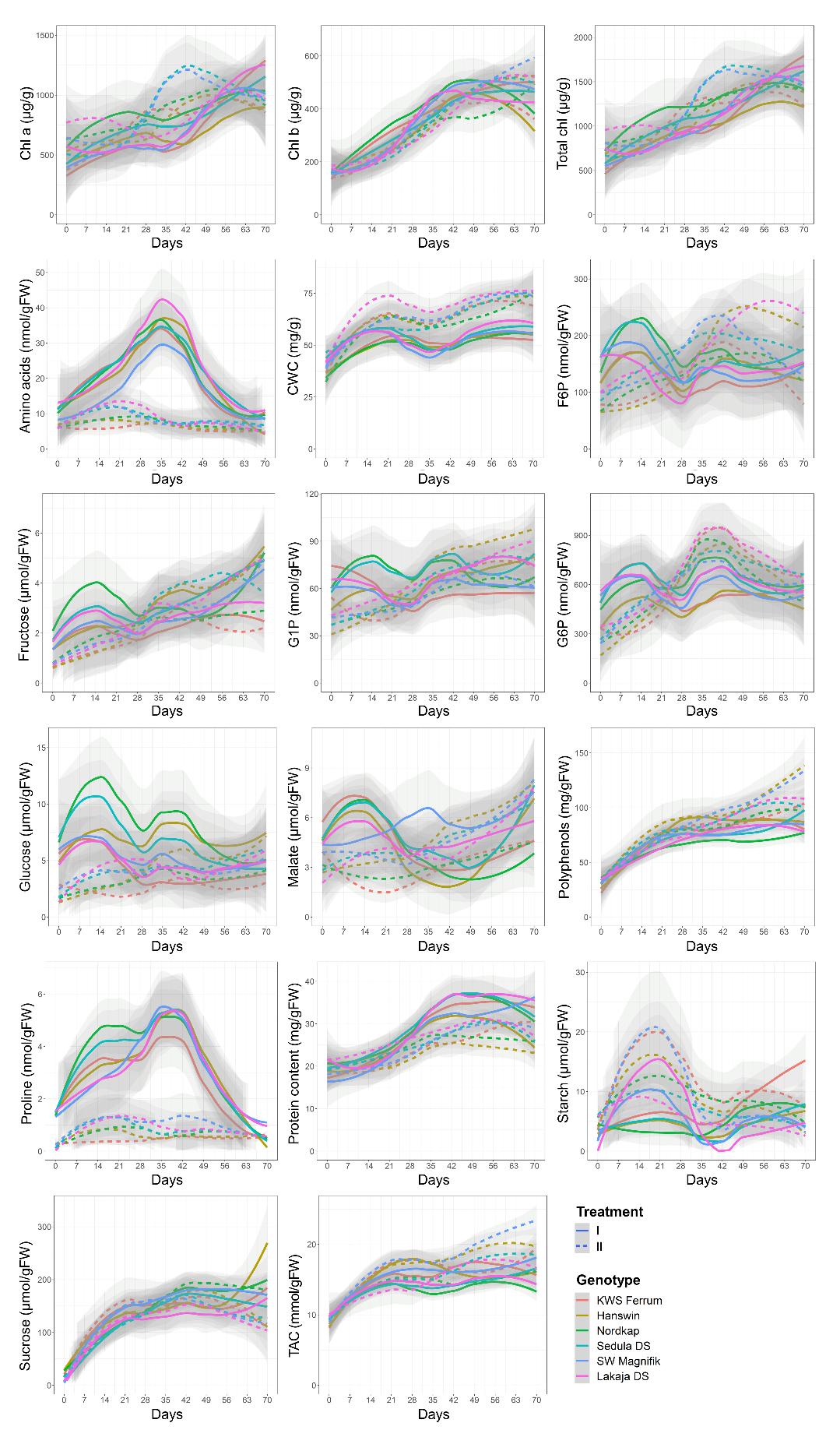


**Supplementary Figure 6.** The effect of temperature during CA on metabolite accumulation in winter wheat leaves. LOESS regression analysis was applied to evaluate the dynamics of 17 different metabolites of 6 winter wheat genotypes during 49 days of CA, 7 days of DEA, and 14 days of REA in two treatment groups. The grey areas indicate the 95% confidence interval.

**Supplementary Table 7.** Kruskal-Wallis test results of differences between concentrations of metabolites in leaf tissues after 49 days of CA, DEA and REA.

| **Treatment** | **Metabolite** | ***X*^2^** | **p-value** | ***Post hoc*** |
| --- | --- | --- | --- | --- |
| I | Chlorophyll a | 17.785 | 0.0001374 *** | CA < DEA = REA |
|  | Chlorophyll b | 3.3387 | 0.1884 | - |
|  | Total chlorophyll | 16.13 | 0.0003144 *** | CA < DEA = REA |
|  | CWC | 4.2128 | 0.1217 | - |
|  | Amino acids | 21.578 | 2.062e-05 *** | CA > DEA = REA |
|  | F6P | 21.926 | 1.733e-05 *** | CA = REA > DEA |
|  | Fructose | 5.377 | 0.06798 | - |
|  | G1P | 2.0557 | 0.3578 | - |
|  | G6P | 16.158 | 0.00031 *** | CA = REA > DEA |
|  | Glucose | 10.465 | 0.005339 ** | CA = REA > DEA |
|  | Malate | 13.24 | 0.001333 ** | CA = DEA < REA |
|  | Polyphenols | 1.487 | 0.4753 | - |
|  | Proline | 37.224 | 8.259e-09 *** | CA > REA > DEA |
|  | Proteins | 6.2182 | 0.04464 * | CA > DEA = REA |
|  | Starch | 24.748 | 4.227e-06 *** | CA < REA < DEA |
|  | Sucrose | 18.263 | 0.0001082 *** | CA = REA > DEA |
|  | TAC | 1.3104 | 0.5193 | - |
| II | Chlorophyll a | 8.4215 | 0.01483 * | CA = REA < DEA |
|  | Chlorophyll b | 4.0193 | 0.134 | - |
|  | Total chlorophyll | 5.6031 | 0.06071 | - |
|  | CWC | 5.1835 | 0.07489 | - |
|  | Amino acids | 10.278 | 0.005864 ** | CA = DEA > REA |
|  | F6P | 13.994 | 0.0009148 *** | CA > DEA = REA |
|  | Fructose | 12.03 | 0.002442 ** | CA = REA > DEA |
|  | G1P | 2.8637 | 0.2389 | - |
|  | G6P | 25.512 | 2.886e-06 *** | CA > DEA = REA |
|  | Glucose | 27.846 | 8.979e-07 *** | CA = REA > DEA |
|  | Malate | 16.349 | 0.0002817 *** | REA > CA > DEA |
|  | Polyphenols | 0.83367 | 0.6591 | - |
|  | Proline | 7.5026 | 0.02349 * | CA ≥ REA ≥ DEA |
|  | Proteins | 3.7733 | 0.1516 | - |
|  | Starch | 27.054 | 1.335e-06 *** | CA = REA < DEA |
|  | Sucrose | 16.297 | 0.0002892 *** | CA > DEA = REA |
|  | TAC | 2.185 | 0.3354 | - |

** indicates significant differences at p < 0.05, ** at p < 0.01, and *** at p < 0.001.*

**Supplementary Table 8.** Kruskal-Wallis test results of the effect of genotype and treatment on metabolite concentration in winter wheat leaves during CA, DEA and REA.

|  |  |  | **CA, day 7** | | **CA, day 49** | | **DEA, day 56** | | **REA, day 70** | |
| --- | --- | --- | --- | --- | --- | --- | --- | --- | --- | --- |
| **Metabolite** | **Factor** | **D.f.** | **H value** | **p-value** | **H value** | **p-value** | **H value** | **p-value** | **H value** | **p-value** |
| Chl a | Genotype | 5 | 6.5796 | 0.2538 | 11.619 | 0.0404 * | 4.1592 | 0.5267 | 5.6547 | 0.3413 |
|  | Treatment | 1 | 11.036 | 0.0008936 *** | 3.973 | 0.04624 * | 3.8478 | 0.04981 * | 3.1391 | 0.07643 |
| Chl b | Genotype | 5 | 4.1742 | 0.5246 | 4.7057 | 0.4528 | 5.5826 | 0.349 | 3.6607 | 0.5992 |
|  | Treatment | 1 | 1.6827 | 0.1946 | 1.9379 | 0.1639 | 0 | 1 | 1.2973 | 0.2547 |
| Total chl | Genotype | 5 | 5.8979 | 0.3163 | 9.8589 | 0.07933 | 3.8378 | 0.573 | 0.44144 | 0.5064 |
|  | Treatment | 1 | 11.036 | 0.0008936 *** | 3.3674 | 0.0665 | 2.027 | 0.1545 | 2.5616 | 0.7672 |
| Protein content | Genotype | 5 | 4.7748 | 0.444 | 8.3363 | 0.1386 | 6.003 | 0.3059 | 12.126 | 0.0331 * |
|  | Treatment | 1 | 13.009 | 0.00031 *** | 11.247 | 0.0007974 *** | 4.9049 | 0.02678 * | 5.6306 | 0.01765 * |
| CWC | Genotype | 5 | 9.8769 | 0.0788 | 5.2657 | 0.3843 | 1.988 | 0.8508 | 1.8679 | 0.8671 |
|  | Treatment | 1 | 1.6016 | 0.2057 | 24.992 | 5.756e-07 *** | 25.626 | 4.145e-07 *** | 25.626 | 4.145e-07 *** |
| TAC | Genotype | 5 | 8.4853 | 0.1314 | 8.0751 | 0.1521 | 7.7267 | 0.172 | 11.844 | 0.03699 * |
|  | Treatment | 1 | 0.056314 | 0.8124 | 7.2322 | 0.007161 ** | 0.84184 | 0.3589 | 7.0631 | 0.007869 ** |
| Polyphenols | Genotype | 5 | 4.6216 | 0.4638 | 3.2042 | 0.6685 | 7.8108 | 0.167 | 4.4324 | 0.489 |
|  | Treatment | 1 | 7.4034 | 0.00651 ** | 15.391 | 8.739e-05 *** | 4.3604 | 0.03678 * | 10.827 | 0.001 ** |
| Amino acids | Genotype | 5 | 0.78979 | 0.9777 | 2.3934 | 0.7925 | 2.5089 | 0.7752 | 5.3346 | 0.3764 |
|  | Treatment | 1 | 21.926 | 2.834e-06 *** | 26.27 | 2.968e-07 *** | 13.242 | 0.0002738 *** | 13.825 | 0.0002006 *** |
| Proline | Genotype | 5 | 0.82249 | 0.9756 | 1.1712 | 0.9476 | 4.3115 | 0.5055 | 1.9327 | 0.8584 |
|  | Treatment | 1 | 26.308 | 2.912e-07 *** | 24.674 | 6.791e-07 *** | 0.030327 | 0.8618 | 3.8513 | 0.04971 * |
| Malate | Genotype | 5 | 0.62479 | 0.9868 | 10.828 | 0.05489 | 8.6557 | 0.1236 | 21.358 | 0.0006932 *** |
|  | Treatment | 1 | 25.313 | 4.875e-07 *** | 6.5411 | 0.01054 * | 3.8622 | 0.04938 * | 2.6043 | 0.1066 |
| Starch | Genotype | 5 | 2.2052 | 0.8201 | 15.861 | 0.007251 ** | 21.982 | 0.0005277 *** | 12.835 | 0.02498 * |
|  | Treatment | 1 | 24.363 | 7.977e-07 *** | 7.6659 | 0.005627 ** | 1.2973 | 0.2547 | 8.2893 | 0.003988 ** |
| Sucrose | Genotype | 5 | 3.1321 | 0.6796 | 6.973 | 0.2227 | 7.0871 | 0.2142 | 6.042 | 0.3021 |
|  | Treatment | 1 | 0.44144 | 0.5064 | 0.004004 | 0.9495 | 0.004004 | 0.9495 | 11.247 | 0.0007974 *** |
| Glucose | Genotype | 5 | 3.2215 | 0.6659 | 12.082 | 0.03368 * | 4.6384 | 0.4616 | 11.779 | 0.03795 * |
|  | Treatment | 1 | 26.277 | 2.958e-07 *** | 1.562 | 0.2114 | 6.7333 | 0.009463 ** | 0.0010011 | 0.9748 |
| Fructose | Genotype | 5 | 3.2801 | 0.6569 | 8.1362 | 0.1489 | 9.2402 | 0.09986 | 11.356 | 0.04475 * |
|  | Treatment | 1 | 25.632 | 4.131e-07 *** | 1.0574 | 0.3038 | 5.1892 | 0.02273 * | 0.87146 | 0.3506 |
| G1P | Genotype | 5 | 0.66967 | 0.9846 | 5.8619 | 0.3199 | 7.5465 | 0.1831 | 12.97 | 0.02366 * |
|  | Treatment | 1 | 25.947 | 3.509e-07 *** | 2.3063 | 0.1288 | 4.3604 | 0.03678 * | 0.96196 | 0.3267 |
| G6P | Genotype | 5 | 4.6787 | 0.4563 | 0.24024 | 0.9986 | 7.8258 | 0.1661 | 6.9429 | 0.2249 |
|  | Treatment | 1 | 25.626 | 4.145e-07 *** | 5.9349 | 0.01484 * | 7.4034 | 0.00651 ** | 0.025025 | 0.8743 |
| F6P | Genotype | 5 | 3.6346 | 0.6031 | 3.958 | 0.5555 | 2.5766 | 0.7649 | 10.288 | 0.06747 |
|  | Treatment | 1 | 18.9 | 1.378e-05 *** | 6.7307 | 0.009477 ** | 10.414 | 0.00125 ** | 1.0901 | 0.2965 |

** indicates significant differences at p < 0.05, ** at p < 0.01, and *** at p < 0.001.*


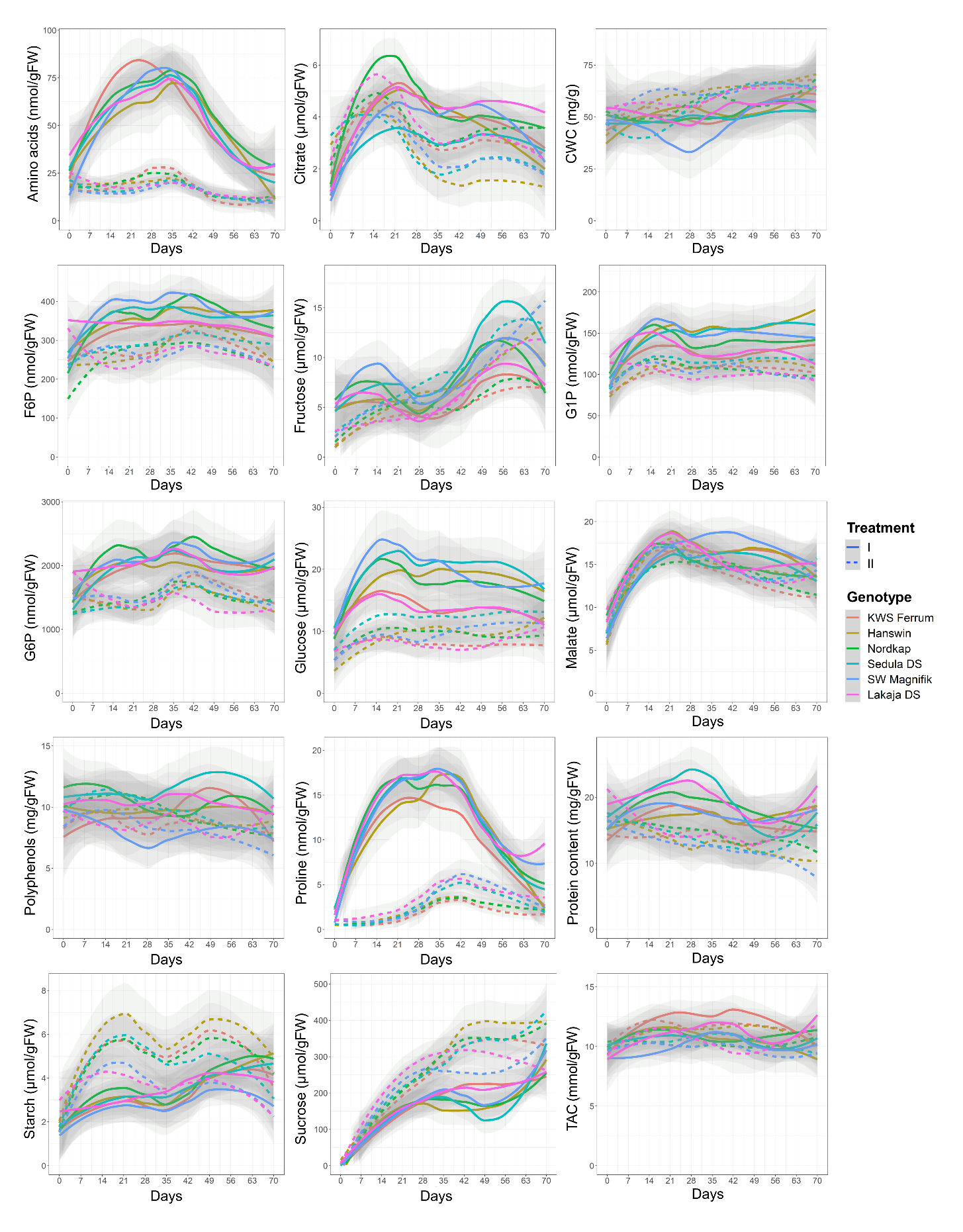


**Supplementary Figure 7.** The effect of temperature during CA on metabolite accumulation in winter wheat crowns. LOESS regression analysis was applied to evaluate the dynamics of 15 different metabolites of 6 winter wheat genotypes during 49 days of CA, followed by 7 days of DEA, and 14 days of REA in two treatment groups. The grey areas indicate the 95% confidence interval.

**Supplementary Table 9.** Kruskal-Wallis test results of differences between concentrations of metabolites in crown tissues after 49 days of CA, DEA and REA.

| **Treatment** | **Metabolite** | ***X*^2^** | **p-value** | ***Post hoc*** |
| --- | --- | --- | --- | --- |
| I | CWC | 2.7531 | 0.2525 | - |
|  | Amino acids | 18.209 | 0.0001112 *** | CA > DEA = REA |
|  | F6P | 12.83 | 0.001637 ** | CA > DEA = REA |
|  | Fructose | 29.053 | 4.912e-07 *** | CA = REA < DEA |
|  | G1P | 3.984 | 0.1364 | - |
|  | G6P | 15.118 | 0.0005214 *** | CA = REA > DEA |
|  | Glucose | 10.738 | 0.004659 ** | CA ≥ DEA ≥ REA |
|  | Citrate | 11.515 | 0.00316 ** | CA = DEA > REA |
|  | Malate | 7.8594 | 0.01965 * | CA ≥ DEA ≥ REA |
|  | Polyphenols | 8.6057 | 0.01353 * | CA = DEA > REA |
|  | Proline | 37.176 | 8.457e-09 *** | CA > REA > DEA |
|  | Proteins | 3.3468 | 0.1876 | - |
|  | Starch | 25.31 | 3.192e-06 *** | CA < REA < DEA |
|  | Sucrose | 26.064 | 2.189e-06 *** | DEA < CA < REA |
|  | TAC | 0.67829 | 0.7124 | - |
| II | CWC | 2.8666 | 0.2385 | - |
|  | Amino acids | 5.3895 | 0.06756 | - |
|  | F6P | 9.591 | 0.008267 ** | CA ≥ DEA ≥ REA |
|  | Fructose | 9.0786 | 0.01068 ** | CA < DEA = REA |
|  | G1P | 1.9479 | 0.3776 | - |
|  | G6P | 7.5165 | 0.02332 * | CA ≥ REA ≥ DEA |
|  | Glucose | 1.552 | 0.4602 | - |
|  | Citrate | 5.436 | 0.06601 | - |
|  | Malate | 1.2853 | 0.5259 | - |
|  | Polyphenols | 2.1854 | 0.3353 | - |
|  | Proline | 26.349 | 1.899e-06 *** | CA > REA > DEA |
|  | Proteins | 2.9675 | 0.2268 | - |
|  | Starch | 23.269 | 8.857e-06 *** | CA = REA < DEA |
|  | Sucrose | 9.9883 | 0.006777 ** | CA = REA > DEA |
|  | TAC | 3.1048 | 0.2117 | - |

**indicates significant differences at p < 0.05, ** at p < 0.01, and *** at p < 0.001.*

**Supplementary Table 10.** Kruskal-Wallis test results of the effect of genotype and treatment on metabolite concentration in winter wheat crowns during CA, DEA and REA.

|  |  |  | **CA, day 7** | | **CA, day 49** | | **DEA, day 56** | | **REA, day 70** | |
| --- | --- | --- | --- | --- | --- | --- | --- | --- | --- | --- |
| **Metabolite** | **Factor** | **D.f.** | **H value** | **p-value** | **H value** | **p-value** | **H value** | **p-value** | **H value** | **p-value** |
| Protein content | Genotype | 5 | 6.4171 | 0.2677 | 3.7819 | 0.5812 | 5.1582 | 0.3969 | 5.8018 | 0.326 |
|  | Treatment | 1 | 2.6155 | 0.1058 | 9.7307 | 0.001812 ** | 6.17 | 0.01299 * | 5.046 | 0.02468 * |
| CWC | Genotype | 5 | 3.9775 | 0.5527 | 2.346 | 0.7995 | 3.0556 | 0.6914 | 8.0734 | 0.1522 |
|  | Treatment | 1 | 0.43573 | 0.5092 | 20.154 | 7.146e-06 *** | 4.1022 | 0.04283 * | 12.56 | 0.0003941 *** |
| TAC | Genotype | 5 | 3.0987 | 0.6848 | 5.1302 | 0.4002 | 12.357 | 0.03021 * | 3.0537 | 0.6917 |
|  | Treatment | 1 | 2.1089 | 0.1464 | 0.027233 | 0.8689 | 0.78478 | 0.3757 | 0.4415 | 0.5064 |
| Polyphenols | Genotype | 5 | 6.4223 | 0.2673 | 4.5875 | 0.4683 | 6.7066 | 0.2434 | 7.9432 | 0.1594 |
|  | Treatment | 1 | 4.2558 | 0.03912 * | 5.8058 | 0.01597 * | 14.537 | 0.0001375 *** | 0.020273 | 0.8868 |
| Amino acids | Genotype | 5 | 2.3362 | 0.8009 | 2.5206 | 0.7734 | 1.6727 | 0.8923 | 2.4865 | 0.7785 |
|  | Treatment | 1 | 20.148 | 7.167e-06 *** | 25.168 | 5.255e-07 *** | 19.34 | 1.094e-05 *** | 25.626 | 4.145e-07 *** |
| Proline | Genotype | 5 | 1.6807 | 0.8913 | 1.6838 | 0.8909 | 6.1472 | 0.2922 | 6.9105 | 0.2274 |
|  | Treatment | 1 | 25.171 | 5.246e-07 *** | 25.5 | 4.424e-07 *** | 11.571 | 0.00067 *** | 20.48 | 6.026e-06 *** |
| Malate | Genotype | 5 | 1.8305 | 0.8721 | 3.0061 | 0.6991 | 2.7924 | 0.732 | 2.3754 | 0.7951 |
|  | Treatment | 1 | 0.21351 | 0.644 | 17.297 | 3.198e-05 *** | 7.5776 | 0.00591 ** | 1.0901 | 0.2965 |
| Citrate | Genotype | 5 | 4.8043 | 0.4402 | 10.596 | 0.06001 | 9.2666 | 0.09889 | 13.684 | 0.01774 * |
|  | Treatment | 1 | 16.752 | 4.26e-05 *** | 13.547 | 0.0002327 *** | 7.8421 | 0.005104 ** | 4.2951 | 0.03822 * |
| Starch | Genotype | 5 | 5.2995 | 0.3804 | 4.5233 | 0.4768 | 14.105 | 0.01496 * | 14.286 | 0.01389 * |
|  | Treatment | 1 | 16.617 | 4.574e-05 *** | 7.7791 | 0.005285 ** | 1.3704 | 0.2417 | 2.6046 | 0.1066 |
| Sucrose | Genotype | 5 | 7.3815 | 0.1938 | 3.3273 | 0.6497 | 3.1291 | 0.6801 | 9.2462 | 0.09964 |
|  | Treatment | 1 | 10.076 | 0.001502 ** | 18.127 | 2.066e-05 *** | 21.337 | 3.852e-06 *** | 10.01 | 0.001557 ** |
| Glucose | Genotype | 5 | 2.2365 | 0.8155 | 5.1654 | 0.396 | 3.0177 | 0.6973 | 8.7087 | 0.1213 |
|  | Treatment | 1 | 22.903 | 1.704e-06 *** | 19.56 | 9.749e-06 *** | 21.929 | 2.83e-06 *** | 10.414 | 0.00125 ** |
| Fructose | Genotype | 5 | 2.1921 | 0.822 | 7.1459 | 0.21 | 7.9943 | 0.1566 | 17.231 | 0.004082 ** |
|  | Treatment | 1 | 25.5 | 4.424e-07 *** | 2.8896 | 0.08915 | 7.4907 | 0.006202 ** | 2.9189 | 0.08755 |
| G1P | Genotype | 5 | 1.9527 | 0.8557 | 5.9441 | 0.3117 | 1.3423 | 0.9305 | 6.018 | 0.3045 |
|  | Treatment | 1 | 7.6863 | 0.005564 ** | 16.48 | 4.916e-05 *** | 19.34 | 1.094e-05 *** | 18.788 | 1.461e-05 *** |
| G6P | Genotype | 5 | 4.9679 | 0.4198 | 0.72349 | 0.9817 | 6.7387 | 0.2408 | 0.74174 | 0.9806 |
|  | Treatment | 1 | 11.333 | 0.0007613 *** | 11.782 | 0.000598 *** | 11.46 | 0.0007109 *** | 18.788 | 1.461e-05 *** |
| F6P | Genotype | 5 | 3.179 | 0.6724 | 2.7746 | 0.7347 | 1.1321 | 0.9512 | 3.4474 | 0.6314 |
|  | Treatment | 1 | 13.91 | 0.0001918 *** | 13.181 | 0.0002828 *** | 8.1081 | 0.004407 ** | 18.515 | 1.686e-05 *** |

** indicates significant differences at p < 0.05, ** at p < 0.01, and *** at p < 0.001.*

**
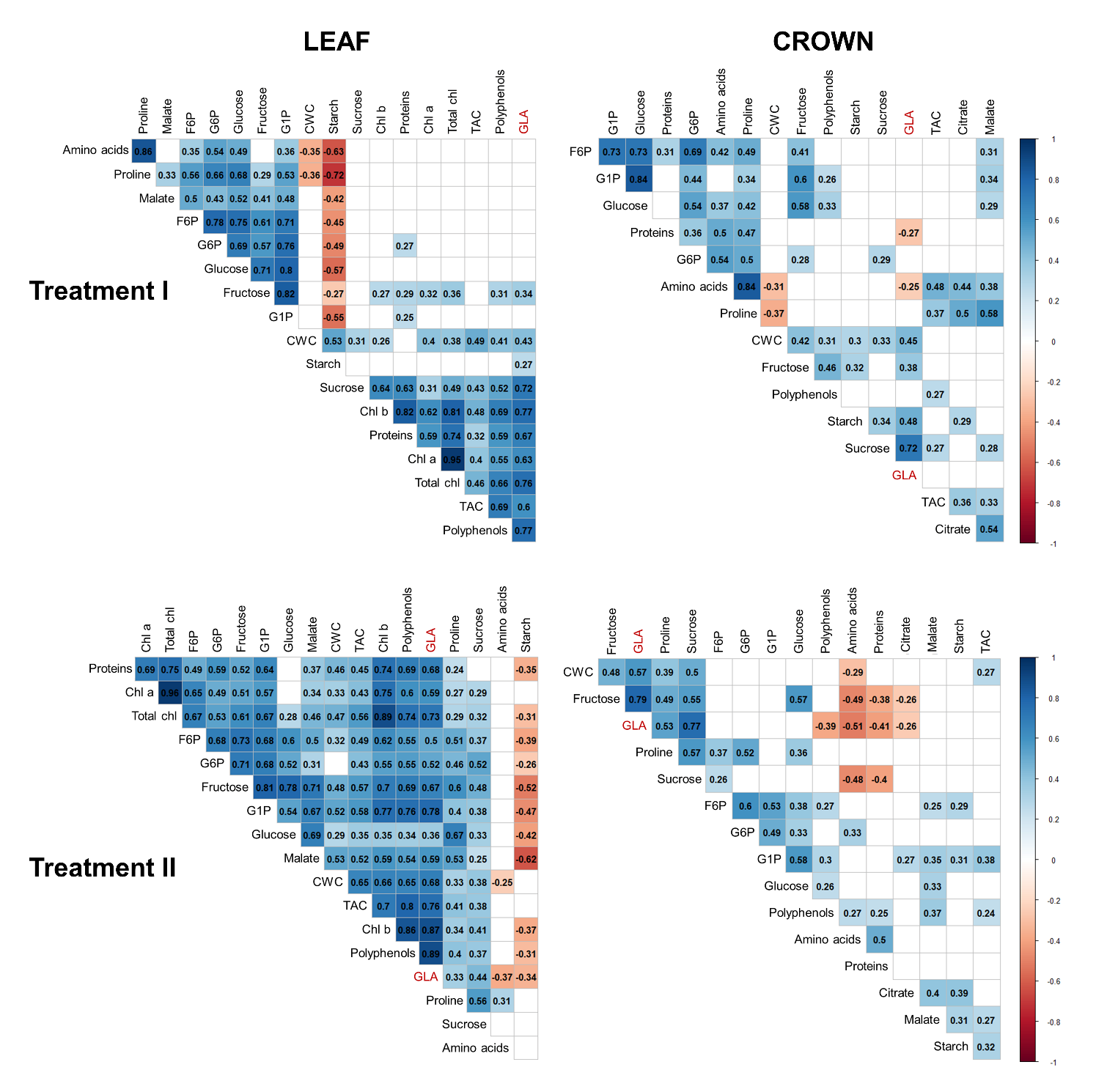
**

**Supplementary Figure 8.** Spearman correlation coefficients (*r*-values) of leaf and crown tissue metabolites against GLA values throughout CA, DEA and REA in treatments I and II. Only statistically significant (p < 0.05) results are shown.


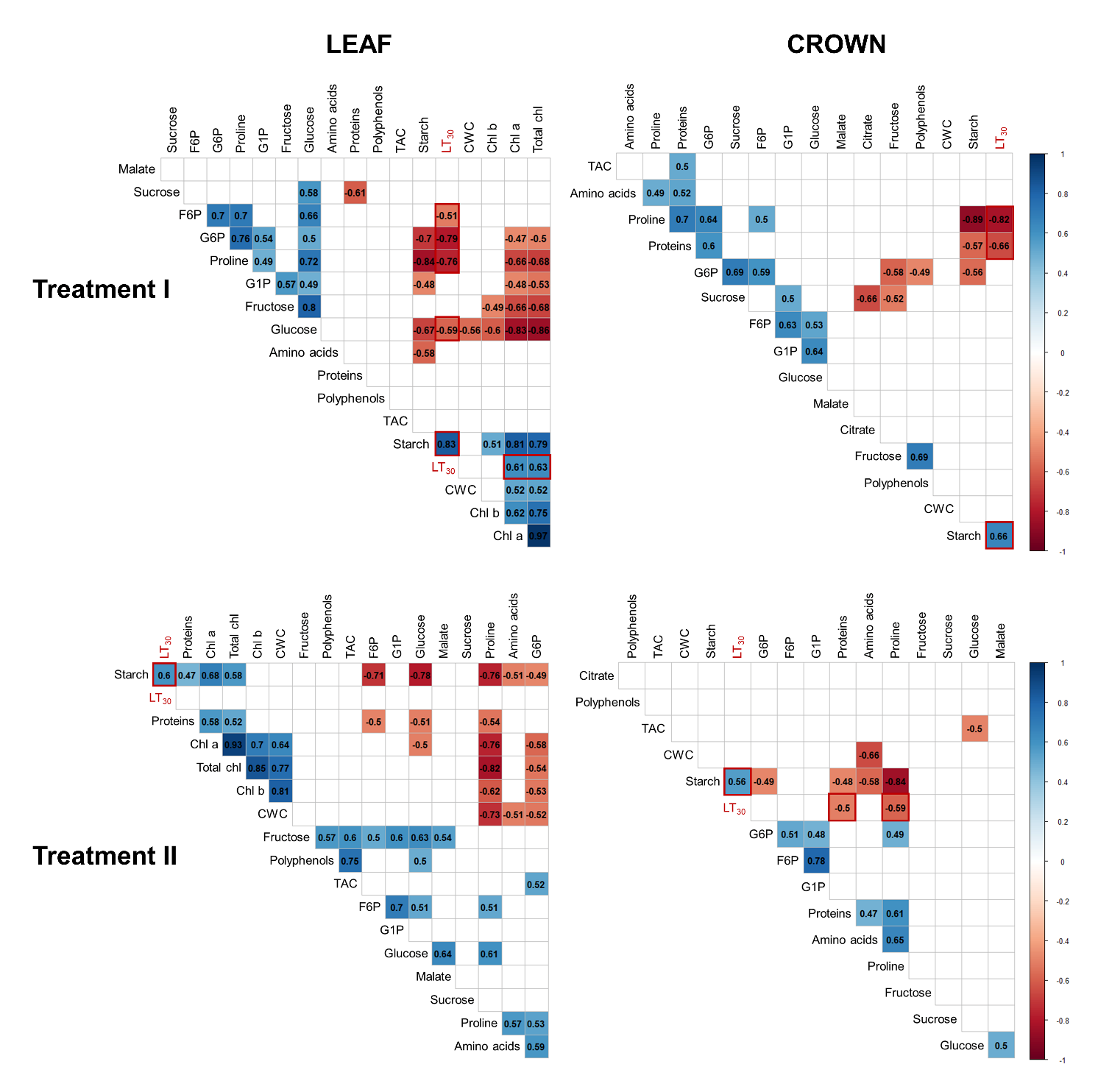


**Supplementary Figure 9.** Spearman correlation coefficients (*r*-values) of leaf and crown tissue metabolites against LT_30_ values throughout CA, DEA and REA in treatments I and II. Only statistically significant (p < 0.05) results are shown. *Note: LT_30_ values are negative, therefore, positive correlations with LT_30_ values ultimately indicate negative correlations with FT, and vice versa.*


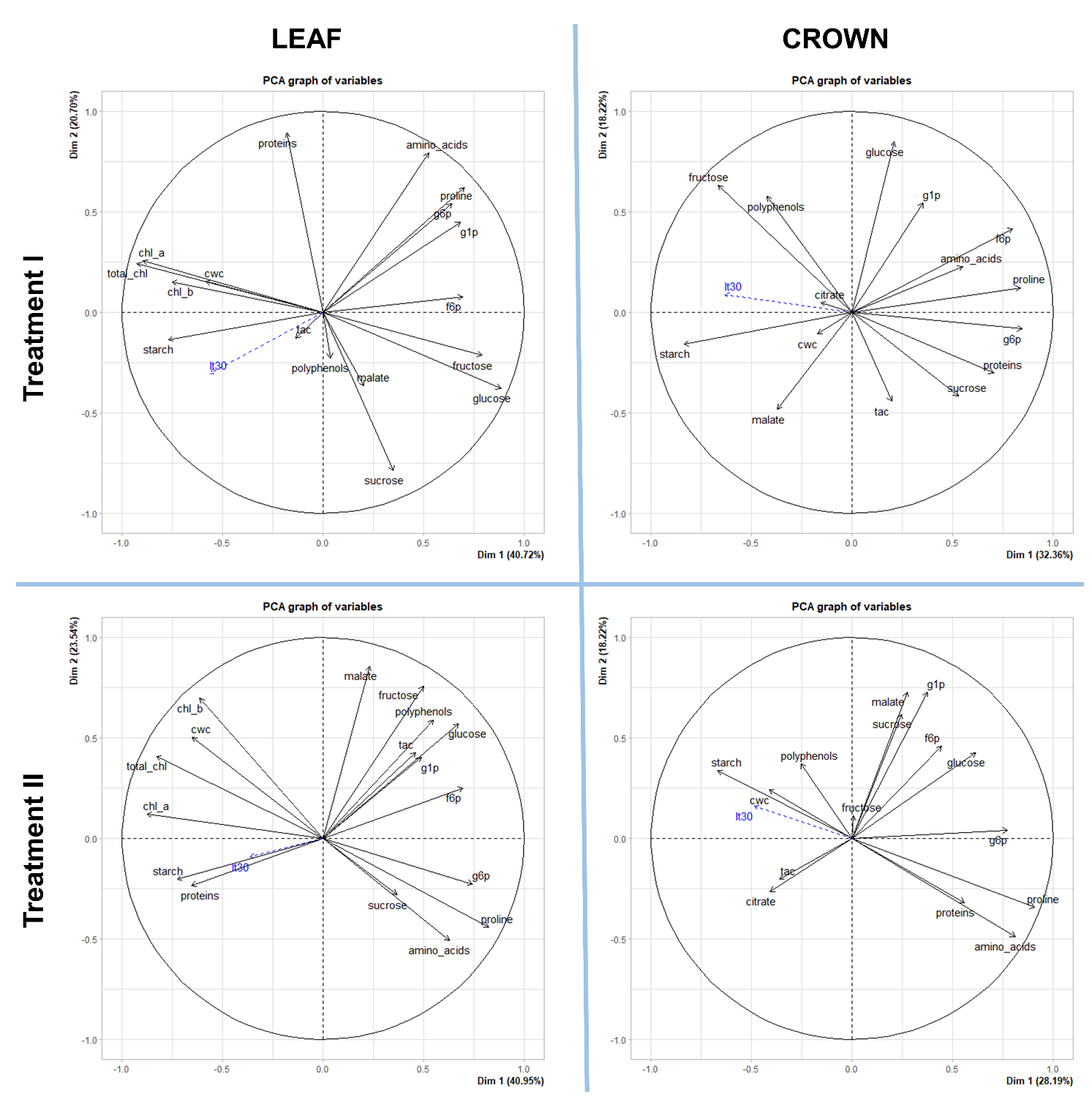


**Supplementary Figure 10.** Principal component analysis (PCA) variable correlation plots of leaf and crown tissue metabolites throughout CA, DEA and REA in treatments I and II with LT_30_ as a supplementary factor. *Note: LT_30_ values are negative, therefore, positive correlations with LT_30_ values ultimately indicate negative correlations with FT, and vice versa.*
